# Engineering of cell-surface receptors for analysis of receptor internalization and detection of receptor-specific glycosylation†

**DOI:** 10.1039/d3sc05054h

**Published:** 2023-12-14

**Authors:** Chang-Hee Lee, Sookil Park, Sanggil Kim, Ji Young Hyun, Hyun Soo Lee, Injae Shin

**Affiliations:** a Department of Chemistry, Yonsei University Seoul 03722 Republic of Korea injae@yonsei.ac.kr; b Department of Chemistry, Sogang University Seoul 04107 Republic of Korea; c Data Convergence Drug Research Center, Korea Research Institute of Chemical Technology Daejeon 34114 Republic of Korea

## Abstract

The epidermal growth factor receptor (EGFR) is a cell-surface glycoprotein that is involved mainly in cell proliferation. Overexpression of this receptor is intimately related to the development of a broad spectrum of tumors. In addition, glycans linked to the EGFR are known to affect its EGF-induced activation. Because of the pathophysiological significance of the EGFR, we prepared a fluorescently labeled EGFR (EGFR128-AZDye 488) on the cell surface by employing the genetic code expansion technique and bioorthogonal chemistry. EGFR128-AZDye 488 was initially utilized to investigate time-dependent endocytosis of the EGFR in live cells. The results showed that an EGFR inhibitor and antibody suppress endocytosis of the EGFR promoted by the EGF, and that lectins recognizing glycans of the EGFR do not enhance EGFR internalization into cells. Observations made in studies of the effects of appended glycans on the entry of the EGFR into cells indicate that a de-sialylated or de-fucosylated EGFR is internalized into cells more efficiently than a wild-type EGFR. Furthermore, by using the FRET-based imaging method of cells which contain an EGFR linked to AZDye 488 (a FRET donor) and cellular glycans labeled with rhodamine (a FRET acceptor), sialic acid residues attached to the EGFR were specifically detected on the live cell surface. Taken together, the results suggest that a fluorescently labeled EGFR will be a valuable tool in studies aimed at gaining an understanding of cellular functions of the EGFR.

## Abstract 2

The genetic code expansion technique was employed to understand functions of the cell-surface receptor.

## Graphical abstract

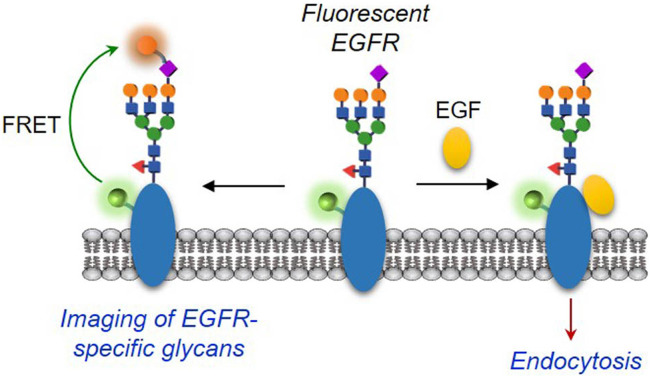

## Introduction

Receptors embedded in plasma membranes of cells transform extracellular signals into intracellular signals through binding to specific ligands outside cells.^1,2^ These cell-surface receptors are heavily modified with glycans to produce glycoproteins by the cooperative action of glycosidases and glycosyltransferases in the endoplasmic reticulum and Golgi apparatus.^3^ Glycans attached to membrane-bound receptors not only profoundly influence their physicochemical properties, such as solubility, stability and folding, but they also impact various cellular functions including ligand binding, oligomerization and signaling pathways.^4–8^ Previous studies have shown that aberrant protein glycosylation is closely associated with a wide range of diseases, particularly cancer.^3,9–12^

**Fig. 1:**
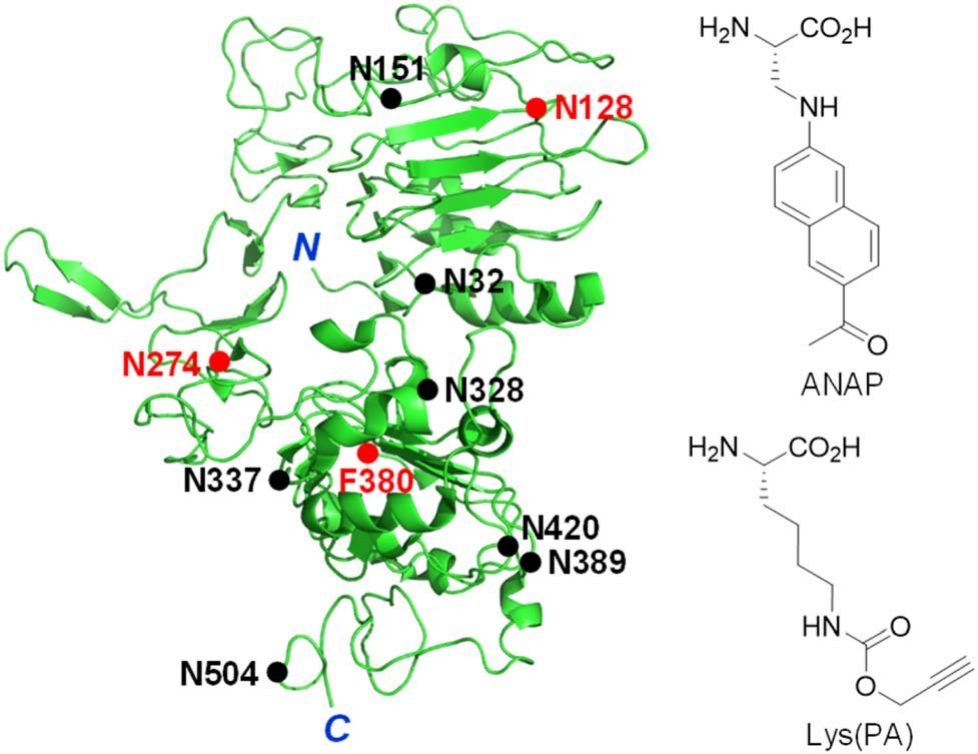
Structure of the truncated extracellular domain of the EGFR (PDB 1IVO). An unnatural amino acid, ANAP or Lys(PA), is incorporated into the EGFR at N128, N274 or F380 positions (red closed circle). *N*-Glycosylated sites are shown as a black closed circle.

The epidermal growth factor receptor (EGFR) is a typical single transmembrane glycoprotein that plays a crucial role in cell proliferation, differentiation and survival.^13–17^ The EGFR is composed of three components, including an extracellular ligand-binding region, a single transmembrane region, and an intracellular region bearing a tyrosine (Tyr) kinase (RTK) domain and a Tyr phosphorylation domain. Following binding of its cognate ligand EGF to the extracellular domain, the EGFR undergoes a conformational change to promote its dimerization and simultaneous activation of its intrinsic Tyr kinase, leading to phosphorylation of Tyr residues.^18^ This event triggers multiple signaling pathways which occur mostly on the plasma membrane. Also, the EGF-activated EGFR is internalized into cells *via* endocytosis to induce signaling pathways.^18^ Over-activation of the EGFR caused by overexpression or mutations takes place in a variety of human tumors, suggesting that this receptor is an important target for the discovery of anticancer agents.^16,17,19,20^ Moreover, this receptor is known to be involved in resistance to chemotherapy and radiation treatment of cancer.^21^ The EGFR is modified with numerous *N*-glycans at asparagine residues of the extracellular domain (Fig. 1),^22,23^ and these modifications affect its ligand-induced dimerization, Tyr phosphorylation and intracellular signaling.^24–26^ Owing to the importance of cellular functions of the EGFR as well as the significant effect of linked glycans on its activity, development of methods for real-time monitoring of EGFR internalization induced by ligands and visualization of EGFR-specific glycosylation are highly important for basic studies and biomedical applications.

Fluorescent proteins have been widely utilized to image specific proteins in live cells and organisms.^27–30^ However, despite their clear benefits of protein imaging, fusion of fluorescent proteins to cell-surface receptors can hamper proper folding of large cell-surface receptors in cells, and may adversely affect ligand binding required for activation as well as subsequent cellular events.^31,32^ To avoid these issues, in the current study the genetic code expansion technique, which enables site-specific insertion of unnatural amino acids (UAAs) into proteins in cells,^33–37^ was applied for construction of fluorescently labeled EGFR on the cell surface. As described below, use of a fluorescent EGFR enabled us to determine the effects of external substances and attached glycans on internalization of the EGFR in live cells. In addition, by FRET-based detection of cells that expresses FRET donor-labeled EGFR and FRET acceptor-conjugated cellular glycans, EGFR-specific glycans were successfully visualized on the live cell surface.

## Results and discussion

### Construction of a fluorescent EGFR on the cell surface

The genetic code expansion technique is powerful and highly useful for protein engineering because of its technical simplicity and applicability to any protein.^33–37^ In particular, this method has been employed to generate fluorescently labeled proteins in cells through pathways involving either direct incorporation of a fluorescent amino acid into target proteins^38–41^ or conjugation of a fluorescent dye to an UAA-incorporated target protein *via* bioorthogonal reactions.^42–47^ The two protein labeling methods were evaluated to determine the better approach for construction of a fluorescently labeled EGFR on the cell surface. Three amino acid residues (N128, N274 and F380) that are located in loop regions of the EGFR were chosen as sites for UAA incorporation (Fig. 1).

Initial studies focused on determining the incorporation efficiency of the fluorescent amino acid ANAP into the three positions of the EGFR indicated above (Fig. S1a†). For this purpose, HeLa cells, stably transfected with pANAP possessing the evolved tRNA_CUA_^EcLeu^ and aminoacyl-tRNA synthetase (AnapRS) genes, were separately transfected with the wild-type (WT) or mutant (N128TAG, N274TAG or F380TAG) EGFR gene in the presence of 50 μM ANAP for 24 h.^39–41^ The cells were then extensively washed with fresh culture media to remove remaining ANAP. Analysis of confocal fluorescence microscopy images of live cells showed that intense ANAP fluorescence in the 410–530 nm range (*λ*_ex_ = 405 nm) arises from the interiors (mostly from the endoplasmic reticulum) but not surfaces of cells transfected with the mutant EGFR regardless of the ANAP incorporation sites (Fig. S2 and S3†). Consequently, the method involving direct incorporation of fluorescent ANAP into the EGFR was not applicable to construction of a fluorescent EGFR on the cell surface.

Next, we assessed the efficacy of a two-step fluorescent labeling technique to produce a fluorescent EGFR on the cell surface. This approach involved the initial insertion of the alkynylated amino acid Lys (PA) into the EGFR and subsequent click-type conjugation of the alkyne group to an azide-appended fluorophore (Fig. S1b†). Toward this end, HeLa cells, stably transfected with a plasmid containing pyrrolysyl-tRNA (tRNA_CUA_^Pyl^) and pyrrolysyl-tRNA synthetase (PylRS) from *Methanosarcina mazei*,^46,48,49^ were transfected with the WT or mutant (N128TAG, N274TAG or F380TAG) EGFR gene in the presence of several concentrations of Lys(PA). The cells were then subjected to copper-catalyzed azide–alkyne cycloaddition (CuAAC) with the azide-appended AZDye 488 dye (AZDye 488-N_3_) which is water soluble and emits green fluorescence with an efficiency that is insensitive to pH over a wide range.

**Fig. 2:**
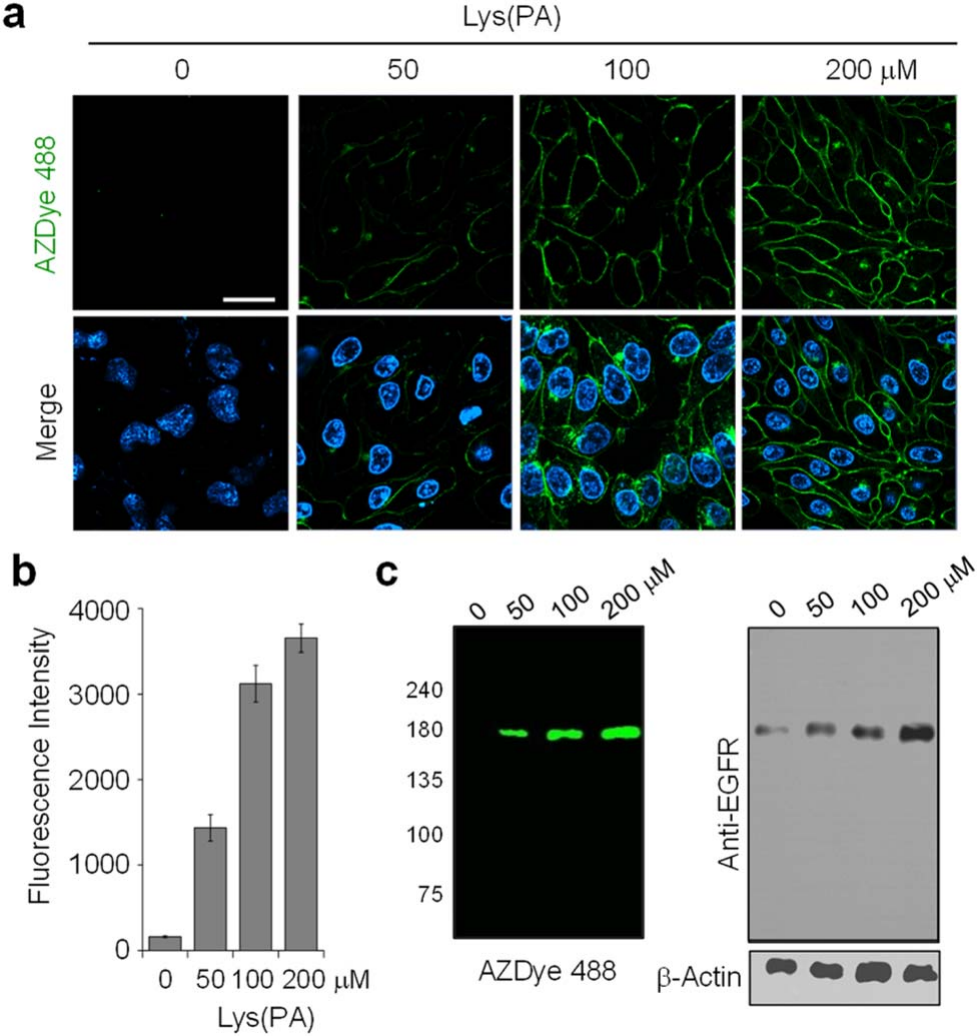
Concentration-dependent incorporation of Lys(PA) into the EGFR. (a) HeLa cells, stably transfected with a plasmid containing a tRNA_CUA_^Pyl^/PylRS pair, were transfected with a mutant EGFR gene (N128TAG) in the presence of several concentrations of Lys(PA) for 48 h. The cells were then subjected to CuAAC with 30 μM AZDye 488-N_3_. The nucleus was stained with Hoechst 33 342. Live cell images were obtained by using confocal fluorescence microscopy (scale bar: 20 μm). Bottom images: merged cell images of Hoechst 33 342 (detected in the 410–460 nm range with excitation at 405 nm) and AZDye 488 fluorescence in the 490–530 nm range (*λ*_ex_ = 488 nm). (b) Graph shows the fluorescence intensity of AZDye 488 in (a) (mean ± s.d., *n* = 3). (c) (Left) Lysates of treated cells in (a) were separated by SDS-PAGE and fluorescently visualized by using a laser scanner. (Right) The expression level of the EGFR was determined by western blot analysis. β-actin was used as a loading control.

As can be seen by inspecting the microscopy images and graphs in Fig. 2 and S3–S5,† incubation of cells, transfected with each mutant gene, with 100–200 μM Lys(PA) for 24–48 h led to efficient generation of fluorescent EGFRs on the cell surface. In particular, transfection of cells with the EGFR-N128TAG gene in the presence of Lys(PA) reproducibly generated slightly more fluorescent EGFRs than that with the other two mutant genes (Fig. S5†). SDS-PAGE gels of cell lysates exhibited a single fluorescence band that corresponded to the EGFR (Fig. 2c). In contrast, cells transfected with the WT EGFR in the presence of Lys(PA) displayed almost no AZDye 488 fluorescence after CuAAC with AZDye 488-N_3_ (Fig. S5†), revealing that background fluorescence arising from nonspecific incorporation of Lys(PA) into proteins is negligible. Thus, the results demonstrate that fluorescent EGFRs can be generated on the cell surface by employing the two-step labeling method.

### Real-time monitoring of endocytosis of the EGFR in live cells

It is known that, upon activation by its cognate ligand, the EGFR is internalized into cells *via* endocytosis.^18^ Despite the biological importance of this process, real-time ligand-induced EGFR internalization in live cells has not been subjected to extensive studies. However, the availability of the AZDye 488-labeled EGFR enabled us to assess the time-dependent endocytosis of the EGFR in live cells. In these experiments, HeLa cells, stably transfected with a plasmid possessing a tRNA_CUA_^Pyl^/PylRS pair, were transfected with the EGFR-N128TAG gene in the presence of 100 μM Lys(PA) for 48 h. The cells were then treated with AZDye 488-N_3_ followed by exposure to LysoTracker Deep Red. After incubation in the presence or absence of the EGF, live cell images were obtained over a 30 min period by using confocal fluorescence microscopy. Internalization of the EGFR into cells was evaluated by determining the percentage of colocalization of AZDye 488 (*λ*_ex_ = 488 nm, detection in the 490–530 nm range) with LysoTracker fluorescence (*λ*_ex_ = 635 nm, detection in the 645–700 nm range).^50–52^

**Fig. 3:**
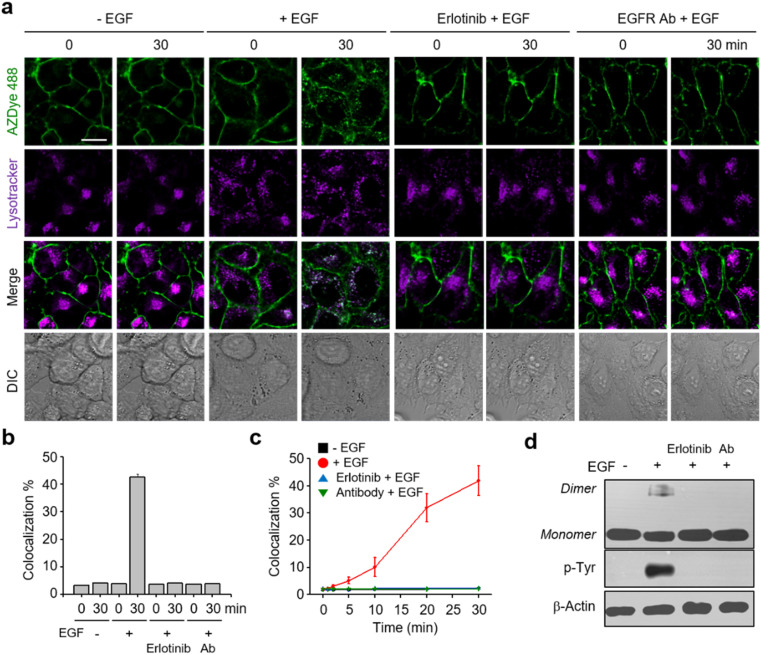
Analysis of endocytosis of the EGFR. (a) HeLa cells containing a tRNA_CUA_^Pyl^/PylRS pair were transfected with an EGFR-N128TAG gene in the presence of 100 μM Lys(PA) for 48 h. The cells were treated with AZDye 488-N_3_ followed by exposure to LysoTracker Deep Red (*λ*_ex_ = 635 nm, detection in the 645–700 nm range). The cells were then incubated for 30 min in the presence or absence of 50 ng mL^−1^ EGF as well as with 50 ng mL^−1^ EGF in the presence of erlotinib or EGFR antibody (EGFR Ab). Live cell images were obtained by using confocal fluorescence microscopy (scale bar: 20 μm). (b) Percentage of colocalization of LysoTracker with AZDye 488 fluorescence, quantified by fluorescence image analysis (mean ± s.d., *n* = 3). (c) Percentage of time-dependent colocalization of LysoTracker with AZDye 488 fluorescence (mean ± s.d., *n* = 3). (d) EGFR dimerization and Tyr phosphorylation of treated cells were analyzed by western blotting.

Analysis of live cell images showed that a gradual time-dependent increase occurs in the colocalization percentage of AZDye 488 with LysoTracker fluorescence when the EGF is present (Fig. 3 and S6–S8†). In addition, the colocalization percentage of AZDye 488 with LysoTracker fluorescence increased in an EGF concentration-dependent manner (Fig. S8a and c†). In contrast, the colocalization percentage was not increased when engineered cells were incubated with the EGF at 4 °C (Fig. S8b and d†). These findings indicate that the EGF enhances endocytosis of the EGFR. To further examine EGFR activation induced by the EGF, the treated cells were subjected to western blot analysis using EGFR and p-Tyr EGFR antibodies. The results showed that EGFR dimerization and Tyr phosphorylation take place only in EGF-treated cells (Fig. 3d).

Following the validation that it could be employed to monitor real-time internalization of the EGFR in live cells, an EGFR labeled with AZDye 488 at the 128 position (EGFR128- AZDye 488) was next used to explore the effects of several external substances, including an EGFR inhibitor, an EGFR antibody and lectins, on EGFR endocytosis. HeLa cells expressing EGFR128-AZDye 488 were treated with the EGF in the presence of either erlotinib (an EGFR tyrosine kinase inhibitor)^53,54^ or EGFR antibody.^55^ Inspection of the fluorescence microscopy images of live cells revealed that co-treatment with the EGF and either erlotinib or antibody does not induce an increase in the colocalization percentage of ADZye 488 with LysoTracker fluorescence, indicating that EGFR endocytosis does not occur (Fig. 3, S9 and S10†). Moreover, dimerization and Tyr phosphorylation of the EGFR did not take place in cells co-treated with the EGF and either an EGFR inhibitor or antibody (Fig. 3d). These observations clearly show that erlotinib and antibody act as blockers for EGF-promoted endocytosis of the EGFR.

**Fig. 4:**
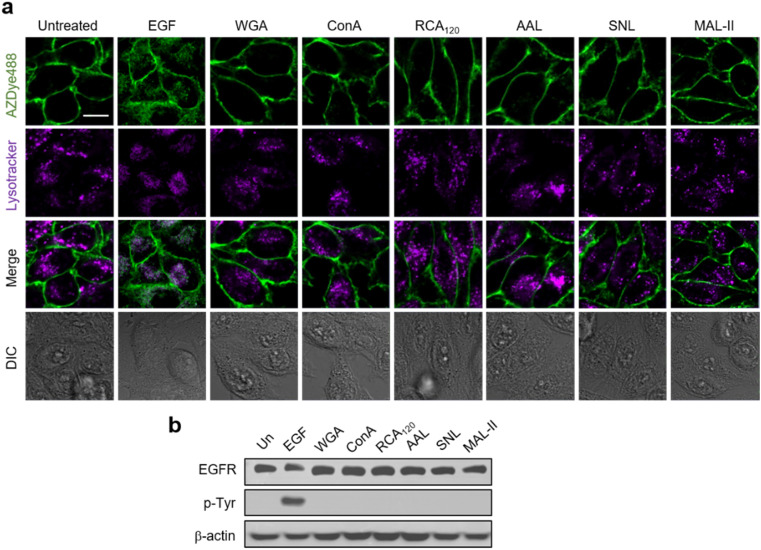
HeLa cells containing a tRNA_CUA_^Pyl^/PylRS pair were transfected with an EGFR-N128TAG gene in the presence of 100 μM Lys(PA) for 48 h. The cells were then subjected to CuAAC with AZDye 488-N_3_. The fluorescently labeled cells were treated with LysoTracker Deep Red followed by exposure to the indicated lectin or EGF as a control. (a) Live cell images were obtained by using confocal fluorescence microscopy (scale bar = 20 μm). (b) EGFR Tyr phosphorylation of treated cells was analyzed by western blotting.

Previous results obtained from immunocytochemistry studies suggested that certain lectins activate the EGFR in the absence of a cognate ligand.^56^ However, detailed investigations of lectin-promoted EGFR activation have not yet been performed. Thus, we utilized EGFR128-AZDye 488 to determine if lectin binding to glycans of the EGFR induces EGFR activation. In these experiments, HeLa cells expressing EGFR128-AZDye 488 were individually incubated in the absence of the EGF with *wheat germ agglutinin* (WGA, GlcNAc binding lectin), *Concanavalin A* (ConA, mannose binding lectin), *Ricinus communis agglutinin I* (RCA_120_, galactose binding lectin), *Aleuria aurantia lectin* (AAL, fucose binding lectin), *Sambucus nigra lectin* (SNL, 2,6-sialic acid binding lectin) or *Maackia amurensis lectin II* (MAL-II, 2,3-sialic acid binding lectin). Analysis of colocalization percentages in live cells showed that unlike treatment with the EGF, none of these lectins promote EGFR internalization into cells (Fig. 4a and S11†). To further ascertain these findings, lysates of treated cells were subjected to western blotting, showing that EGFR Tyr phosphorylation is not generated in cells treated with each lectin (Fig. 4b).

The negative response of lectins on EGFR activation could be caused by a lack of binding to glycans linked to the EGFR. To test this possibility, HeLa cells expressing EGFR128-AZDye 488 were incubated with each of the Cy3-labeled lectins and then LysoTracker Deep Red. We anticipated that the interaction of Cy3-lectin with glycans attached to EGFR128-AZDye 488 would lead to the appearance of FRET signals from AZDye 488 to the Cy3 fluorophore on the cell surface. Indeed, analysis of live cell images showed that FRET-induced fluorescence occurs on the cell surface (Fig. S12†), indicating that lectins bind to glycans of the EGFR. In addition, the observed high colocalization percentage of Cy3 with LysoTracker fluorescence showed that lectins are internalized inside cells, findings that are consistent with those made previously.^57–59^ Collectively, the studies show that although lectins interact with glycans linked to the EGFR, they do not promote endocytosis of the EGFR presumably owing to their inability to induce a conformational change for EGFR activation.

It was suggested earlier that sialylation or fucosylation of the EGFR influences its dimerization and activation.^24^ To explore this feature more fully, EGFR128-AZDye 488 was used to evaluate the effects of glycans attached to the EGFR on endocytosis. Cells expressing EGFR128-AZDye 488 were treated with either fucosidase or sialidase for 1 h to remove the respective cell-surface sialic acid or fucose moieties. To determine the efficiency for the removal of sialic acid and fucose from cell surfaces by the corresponding glycosidases, lysates of glycosidase-treated cells were subjected to immunoprecipitation with EGFR antibody followed by lectin blotting with SNL or AAL. The results showed that treatment with sialidase or fucosidase leads to almost complete degradation of sialic acid or fucose moieties, respectively, from glycans of the EGFR (Fig. S13†).

**Fig. 5:**
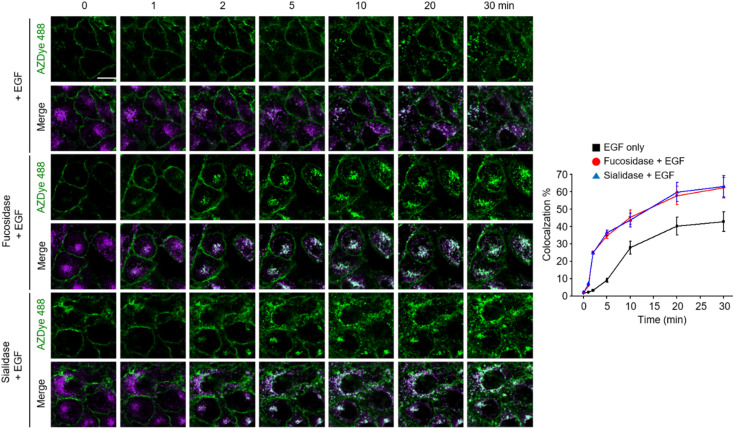
HeLa cells containing a tRNA_CUA_^Pyl^/PylRS pair were transfected with an EGFR-N128TAG gene in the presence of 100 μM Lys(PA) for 48 h. The cells were then treated with AZDye 488-N_3_. The fluorescently labeled cells were incubated with either fucosidase or sialidase for 1 h and then exposed to LysoTracker Deep Red. Live cell images were collected at selected times following the incubation of the EGF by using confocal fluorescence microscopy (scale bar: 20 μm). The graph shows the percentage of time-dependent colocalization of AZDye 488 with LysoTracker fluorescence (mean ± s.d., *n* = 3).

**Fig. 6:**
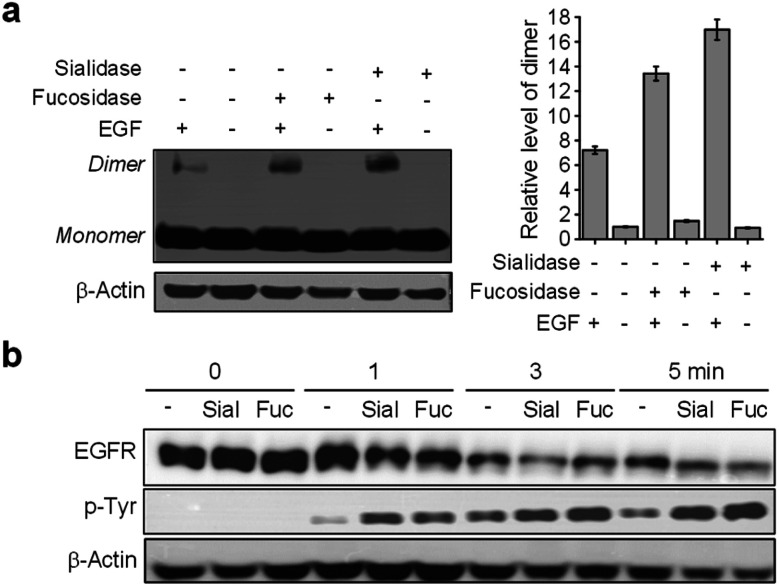
HeLa cells transfected with a tRNA_CUA_^Pyl^/PylRS pair were transfected with an EGFR-N128TAG gene in the presence of 100 μM Lys(PA) for 48 h. The cells were incubated with either fucosidase or sialidase for 1 h and then treated with or not treated with 50 ng mL^−1^ EGF. (a) EGFR dimerization of sialidase- or fucosidase-treated cells was analyzed by western blotting. Graphs show the relative level of an EGFR dimer in treated cells. (b) EGFR Tyr phosphorylation of sialidase- or fucosidase-treated cells was analyzed by western blotting.

Images of live cells were then collected over a 30 min period after incubation of the fucosidase- or sialidase-treated cells, expressing EGFR128-AZDye 488, with the EGF and LysoTracker Deep Red. Analysis of time-dependent colocalization of AZDye 488 with LysoTracker fluorescence in cells revealed that a de-sialylated or de-fucosylated EGFR is internalized into cells more efficiently than an intact EGFR (Fig. 5). Also, a determination of the relative levels of EGFR dimerization and Tyr phosphorylation showed that, compared to untreated controls, sialidase or fucosidase-treated cells display higher levels of EGF-induced EGFR dimerization and Tyr phosphorylation (Fig. 6). This observation is consistent with the results of previous studies,^24^ and reinforces the notion that alterations of glycosylation patterns induced by treatment of sialidase and fucosidase enhance EGF-induced EGFR activation.

### FRET-based detection of cell-surface EGFR-specific glycans

**Fig. 7:**
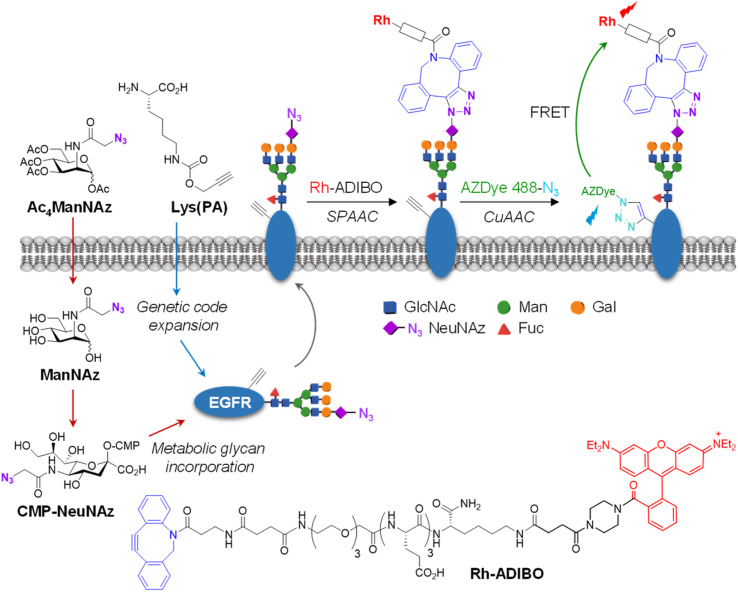
FRET-based detection of cell-surface EGFR-specific glycosylation. Lys(PA) is inserted into the cell-surface EGFR using the genetic code expansion technique. An azido sugar Ac_4_ManNAz is incorporated into cellular glycans through the metabolic glycan process. Specifically, after Ac_4_ManNAz enters cells, it is deacetylated by nonspecific esterases in the cytosol. The resultant ManNAz undergoes a series of enzymatic transformations to generate CMP-NeuNAz. This activated sugar is attached to elongating glycans of glycoconjugates by the action of sialyltransferases. The sialylated glycoconjugates are translocated to the plasma membrane (see the text for detailed explanation).

For visualization of cell-surface receptor-specific glycosylation, several methods, including the *in situ* proximity ligation assay using two different antibodies against a protein and a glycan,^60^*cis*- or transmembrane fluorescence resonance energy transfer (FRET)-based methods,^61,62^ a two-photon fluorescence lifetime imaging method,^63^ and the proximity-induced hybridization chain reaction,^64^ have been developed to date.^65^ In this study, we designed experiments to use an AZDye 488-labeled EGFR for specific detection of glycans linked to the EGFR on the cell surface. However, imaging using a fluorescent EGFR was not effective for visualization of specific glycans of the EGFR. To achieve this goal, we combined a genetic code expansion technique capable of protein-targeted fluorescence imaging with a metabolic glycan incorporation technique that enables fluorescence labeling of the glycan.^66–68^ In this approach, Lys (PA) was inserted into a cell-surface EGFR using the genetic code expansion technique and an azido sugar Ac_4_ManNAz that has been widely used for metabolic glycan labeling studies was concurrently incorporated into cellular glycans through the process shown in Fig. 7.^69,70^ The azide-bearing glycans were then conjugated using strain-promoted azide–alkyne cycloaddition (SPAAC) with cyclooctyne-linked rhodamine (Rh-ADIBO) as a FRET acceptor that contained three glutamates to increase water-solubility and to minimize its entry inside cells.^71^ Also, Lys (PA) of the EGFR was conjugated with FRET donor-containing AZDye 488-N_3_. Consequently, it was anticipated that, upon excitation of AZDye 488, FRET would take place from EGFR-AZDye 488 to rhodamine-labeled glycans of the EGFR in cells (Fig. S14†), which would enable selective detection of glycans attached to the EGFR.

**Fig. 8:**
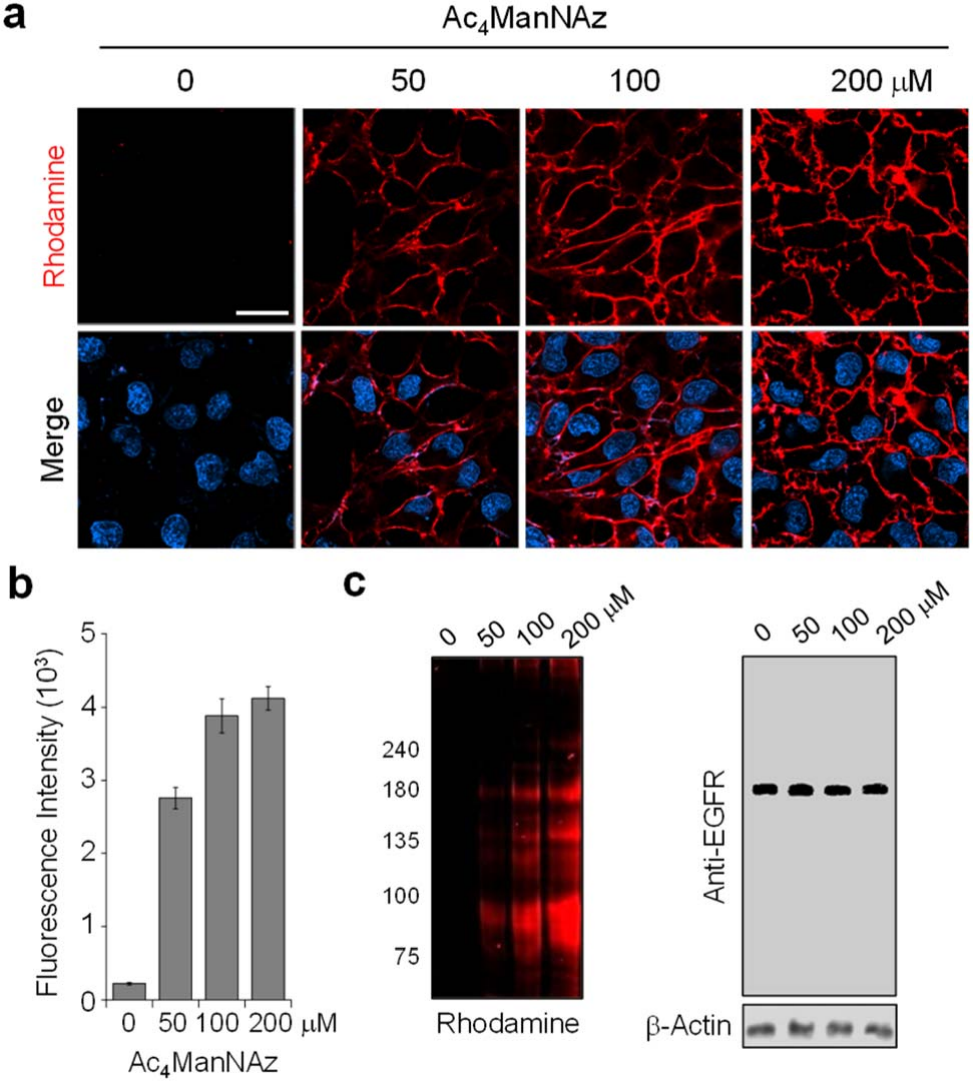
Concentration-dependent metabolic incorporation of Ac_4_ManNAz into cellular glycans. (a) HeLa cells were incubated with several concentrations of Ac_4_ManNAz for 48 h and then treated with Rh-ADIBO. Live cell images were obtained by using confocal fluorescence microscopy (scale bar: 20 μm). Bottom images: merged cell images of Hoechst 33 342 and rhodamine fluorescence (detected in the 570–630 nm range with excitation at 561 nm). (b) Graph shows the fluorescence intensity of rhodamine in (a) (mean ± s.d., *n* = 3). (c) (Left) Lysates of treated cells in (a) were separated by SDS-PAGE and fluorescently visualized by using a laser scanner. (Right) The expression level of the EGFR was analyzed by western blotting.

To optimize metabolic glycan labeling conditions for introduction of the FRET acceptor, HeLa cells were incubated for 48 h with various concentrations of Ac_4_ManNAz, which should be processed by cellular machinery and incorporated into glycoconjugates in cells (Fig. 7). In parallel experiments, cells were also treated with 100 μM Ac_4_ManNAz during several time periods. As a control, cells were exposed to the peracetylated natural sugar Ac_4_ManNAc (100 μM) for 48 h. A subsequent bioorthogonal reaction with Rh-ADIBO was conducted to fluorescently label the cellular glycans. As can be seen in the images and graphs in Fig. 8 and S15,† cells treated with 100 μM Ac_4_ManNAz for 48 h displayed strong rhodamine fluorescence on the cell surface, revealing that an azido sugar is efficiently metabolically incorporated into cellular glycans under these incubation conditions. In contrast, cells incubated with Ac_4_ManNAc did not exhibit rhodamine fluorescence on the cell surface, indicating the importance of an azide functionality for labeling with Rh-ADIBO (Fig. S15†).

Having demonstrated that individual cell-surface EGFRs and glycans could be labeled with the FRET donor and acceptor, respectively, by utilizing two different incorporation methods, we next evaluated the detection efficiency of EGFR-specific glycans in live cells through intramolecular FRET from AZDye 488 to rhodamine upon excitation at 488 nm. HeLa cells, stably transfected with a plasmid containing a tRNA_CUA_^Pyl^/PylRS pair, were transfected with the EGFR gene (WT, N128TAG, N274TAG or F380TAG) in the presence of both 100 μM Lys(PA) and 100 μM Ac_4_ManNAz for 48 h. The incubation conditions did not affect cell viability (Fig. S16†). For glycan and EGFR labeling purposes, the cells were exposed sequentially to Rh-ADIBO and AZDye 488-N_3_, respectively. By means of confocal fluorescence microscopy, the fluorescence of EGFR-AZDye 488 was detected in live cells in the 490–530 nm range with excitation at 488 nm, and that of rhodamine-labeled glycans was monitored in the 570–630 nm range with excitation at 561 nm. In addition, the FRET ratios (*I*_Rh_/*I*_AZDye_), where *I*_Rh_ and *I*_AZDye_ are the respective fluorescence intensities of rhodamine in the 570–630 nm range and AZDye 488 in the 490–530 nm range with excitation at 488 nm, were determined.

**Fig. 9:**
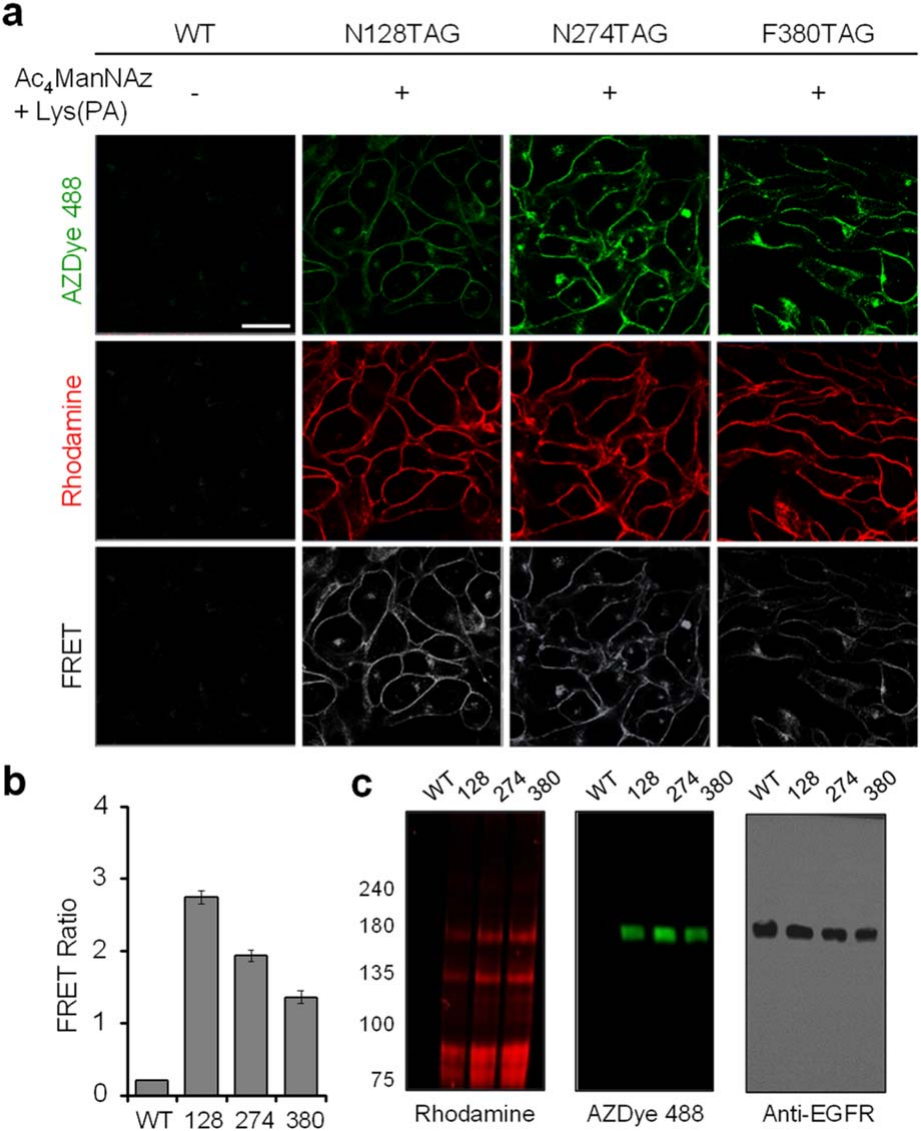
FRET-based detection of EGFR-specific glycans. (a) HeLa cells, stably transfected with a plasmid containing a tRNA_CUA_^Pyl^/PylRS pair, were transfected with an EGFR gene in the presence of both 100 μM Lys(PA) and 100 μM Ac_4_ManNAz for 48 h. The cells were then exposed sequentially to Rh-ADIBO and AZDye 488-N_3_. Live cell images were obtained by using confocal fluorescence microscopy (scale bar: 20 μm). Fluorescence of EGFR-AZDye 488 was detected in live cells in the 490–530 nm range (*λ*_ex_ = 488 nm), and that of rhodamine-labeled glycans was monitored in the 570–630 nm range (*λ*_ex_ = 561 nm). FRET signals were monitored in the 570–630 nm range with excitation of AZDye 488 at 488 nm. (b) Graph displays the FRET ratio (*I*_Rh_/*I*_AZDye_), recorded from live cell images in (a), where *I*_Rh_ and *I*_AZDye_ are the fluorescence intensities of rhodamine in the 570–630 nm range and AZDye 488 in the 490–530 nm range with excitation at 488 nm (mean ± s.d., *n* = 3). (c) Cell lysates were separated by SDS-PAGE and fluorescently visualized by using a laser scanner. The expression level of the EGFR was analyzed for a loading control and protein size verification.

Quantitative analysis of the live cell images indicates that FRET signals arising from the surface of cells transfected with the EGFR-N128TAG gene in the presence of both Lys(PA) and Ac_4_ManNAz are stronger than those from the surface of cells transfected with the EGFR-N274TAG or EGFR-F380TAG gene under the same conditions (Fig. 9). In addition, AZDye 488 fluorescence in cells transfected with an EGFR-N128TAG gene was the weakest among those coming from the three mutant genes. We assumed that AZDye 488 at the 128 position of the EGFR is relatively closer to rhodamine attached to N-glycan sites than the other two positions and, thus, FRET between AZDye 488 at the 128 position of the EGFR and rhodamine takes place more efficiently than that between AZDye 488 at the other position of the EGFR and rhodamine.

In contrast, cells transfected with the EGFR-N128TAG gene in the presence of either Lys(PA) or Ac_4_ManNAz displayed only AZDye 488 or rhodamine fluorescence, respectively, but they did not exhibit FRET signals (Fig. S17†). Fluorescence emission spectra (*λ*_ex_ = 488 nm) of cells transfected with an EGFR-N128TAG gene in the presence or absence of Lys(PA) and Ac_4_ManNAz followed by AZDye 488 and rhodamine labeling were also recorded. An emission band at *ca.* 600 nm, corresponding to the FRET-induced fluorescence, was clearly seen in cells treated with both Lys(PA) and Ac_4_ManNAz (Fig. S18†). However, negligible FRET signals arose in cells treated with Lys(PA) or Ac_4_ManNAz alone. The above results provide clear evidence that, by choosing a FRET pair with well-separated excitation spectra, bleed-through in the FRET channel is satisfactorily suppressed even in the presence of large excess of the FRET acceptor (rhodamine-labeled glycans) on cell surfaces.

**Fig. 10:**
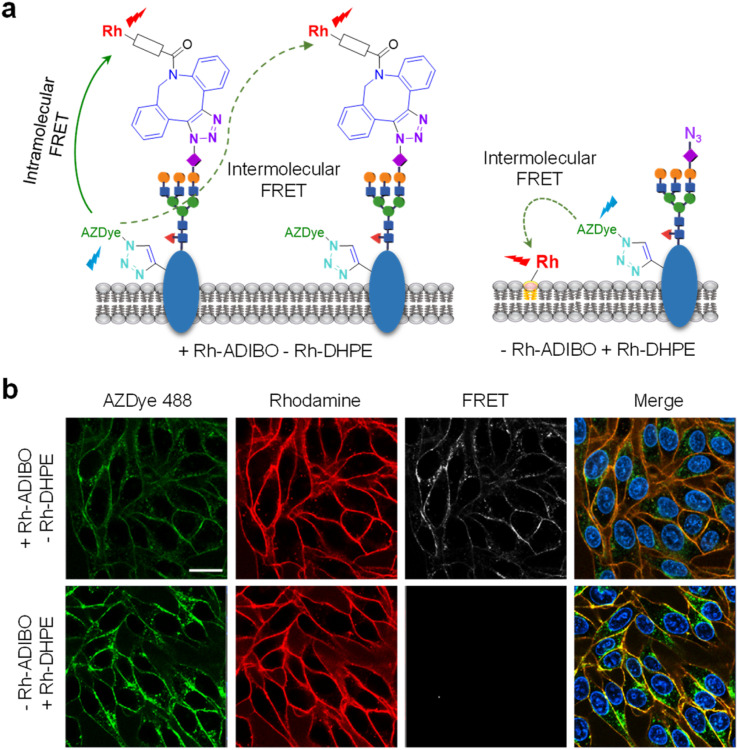
(a) Schematic representation of (left) intramolecular and intermolecular FRET between AZDye 488 and Rh-ADIBO and (right) intermolecular FRET between AZDye 488 and Rh-DHPE in engineered cells. (b) HeLa cells, stably transfected with a plasmid containing a tRNA_CUA_^Pyl^/PylRS pair, were transfected with an EGFR-N128TAG gene in the presence of 100 μM Lys(PA) and 100 μM Ac_4_ManNAz for 48 h. The cells were then treated with (upper images) Rh-ADIBO followed by reaction with AZDye 488-N_3_ and (lower images) Rh-DHPE followed by reaction with AZDye 488-N_3_. Live cell images were obtained by using confocal fluorescence microscopy (scale bar: 20 μm).

We further determined if the observed FRET signals arise from glycans attached directly to the EGFR (intramolecular FRET) or from nearby glycoconjugates (intermolecular FRET) (Fig. 10a, left). For this purpose, HeLa cells, stably transfected with a plasmid containing a tRNA_CUA_^Pyl^/PylRS pair, were transfected with the EGFR-N128TAG gene in the presence of 100 μM of Lys(PA) and 100 μM Ac_4_ManNAz for 48 h. The cells were treated with Rh-DHPE (rhodamine-labeled 1,2-dihexadecanoyl-*sn*-glycero-3-phosphoethanolamine), which inserts uniformly into the cell membrane,^72^ and then subjected to AZDye 488 labeling. We reason that if FRET from AZDye 488 to rhodamine takes place in these cells, it would occur intermolecularly from EGFR-AZDye 488 to nearby Rh-DHPE (Fig. 10a, right). Analysis of live cell images showed that FRET-induced fluorescence from AZDye 488 to Rh-DHPE does not take place on the cell surface (Fig. 10b and S19†). The findings indicate that FRET takes place from EGFR-AZDye 488 to rhodamine in its conjugated glycans, and that nearby glycoconjugates do not influence FRET originating from the EGFR.

In the final phase of this study, we determined whether FRET in the cells is a sialylation state-dependent phenomenon because ManNAz is converted largely to NeuNAz through the metabolic glycan process. HeLa cells containing an EGFR-N128TAG gene were incubated with Lys(PA) and Ac_4_ManNAz for 48 h, and then exposed to sialidase or fucosidase to cleave sialic acid or fucose residues, respectively, from cell-surface glycoconjugates. The cells were then subjected to labeling with Rh-ADIBO and AZDye 488-N_3_. As shown in Fig. S20,† sialidase treatment abolished both rhodamine fluorescence and FRET-induced fluorescence, but fucosidase treatment did not, revealing that sialic acid residues on the EGFR are critical for the observed FRET. Collectively, the findings demonstrate that sialic acid residues conjugated to the EGFR in live cells can be specifically detected by using the current FRET-based imaging method that involves incorporation of a UAA and unnatural sugar into the EGFR and cellular glycans, respectively, followed by labeling with the FRET donor and acceptor.

## Conclusions

In the study described above, a fluorescently labeled EGFR was reproducibly and reliably generated on the cell surface by using the genetic code expansion technology and bioorthogonal chemistry. A fluorescent EGFR, EGFR-AZDye 488, was then applied for time-dependent monitoring of endocytosis of the EGFR in live cells. The results from this effort indicate that an EGFR Tyr kinase inhibitor (erlotinib) and EGFR antibody block EGF-promoted endocytosis of the EGFR. In addition, we observed that lectins recognizing glycans of the EGFR do not stimulate EGFR internalization into cells. Furthermore, the results of an effort aimed at employing EGFR-AZDye 488 to evaluate the effects of glycans linked to the EGFR on its internalization into cells provide clear evidence that sialic acid and fucose residues of glycans conjugated to the EGFR retard EGF-induced endocytosis. Finally, by using the FRET-based imaging method of cells which possess EGFR-AZDye 488 (a FRET donor) and glycans labeled with rhodamine (a FRET acceptor), sialic acid residues conjugated to the EGFR were specifically visualized on the cell surface. Taken together, the results suggest that the fluorescently labeled EGFR constructed by employing the genetic code expansion technology and bioorthogonal chemistry will be a powerful tool in studies aimed at gaining a deeper understanding of the cellular functions of the EGFR.

## Data availability

Additional experimental data supporting this article are included in the ESI.

## Author contributions

I. S. and C.-H. L. designed the study and wrote the paper. I. S. and H. S. L. supervised the project. C.-H. L., S. P., S. K., and J. Y. H. carried out the experiments and analyzed the results. All authors discussed the results and commented on the manuscript.

## Conflicts of interest

There are no conflicts to declare.

## Supplementary Material

SC-015-D3SC05054H-s001
