# Supplementary material for: Engineering of cell-surface receptors for analysis of receptor internalization and detection of receptor-specific glycosylation
Source: Chem Sci. 2023 Dec 14;15(2):555–65. doi: 10.1039/d3sc05054h (PMC10762726; doi:10.1039/d3sc05054h)

Electronic Supplementary Material (ESI) for Chemical Science.
This journal is © The Royal Society of Chemistry 2023

SUPPORTING INFORMATION

Engineering of cell-surface receptors for analysis of receptor internalization and detection of receptor-specific glycosylation

Chang-Hee Lee,[1] Sookil Park,[1] Sanggil Kim,[2] Ji Young Hyun,[3] Hyun Soo Lee,[2] Injae Shin[1,*]

[1] Department of Chemistry, Yonsei University, Seoul 03722, Republic of Korea.

[2] Department of Chemistry, Sogang University, Seoul 04107, Republic of Korea.

[3] Data Convergence Drug Research Center, Korea Research Institute of Chemical Technology, Daejeon 34114, Republic of Korea.

Table of Contents

1. Synthesis …..…………………………………………………………… S2
2. Biology ……..……………………………………………………………… S7
3. Supplementary References ……………………………………………………… S12
4. Supplementary Figures ………………………………………………………. S13
5. NMR spectra ..……………………………………………………………… S33

Synthesis

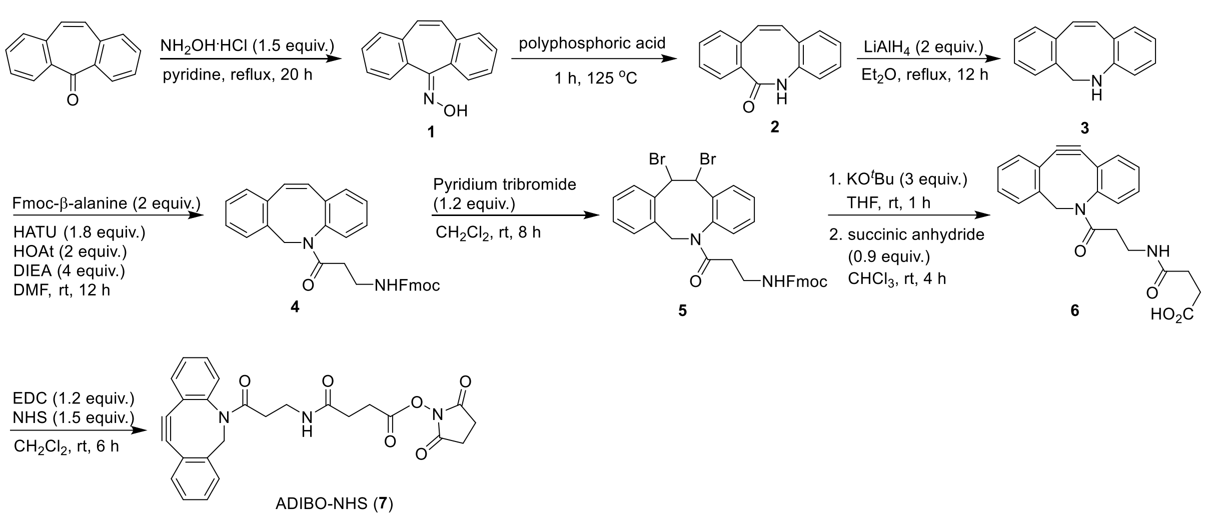

Dibenzosuberenone oxime (**1**, ref.1). To a stirred solution of dibenzosuberenone (25 g, 121 mmol, 1 equiv.) in pyridine (70 mL) was added hydroxylamine hydrochloride (11.5 g, 164 mmol, 1.5 equiv.) at room temperature. The mixture was heated under reflux for 20 h. The mixture was concentrated under reduced pressure and the residue was poured into cold 5% aqueous HCl with crushed ice. After stirring for 20 min at 0 °C, the solid was filtered, washed with cold water and dried under vacuum to provide crude dibenzosuberenone oxime (**1**) as a white solid in 93% yield (25 g, 113 mmol): ^{1}H NMR (400 MHz, $CDCl_3$) δ 9.37 (br. s., 1 H), 7.66-7.64 (m, 1 H), 7.58-7.56 (m, 1 H), 7.43-7.32 (m, 6 H), 6.92 (d, J = 4.8 Hz, 2 H); ^{13}C NMR (100 MHz, $CDCl_3$) δ 156.5, 135.4, 134.5, 133.8, 130.8, 130.6, 130.5, 129.4, 129.2, 129.0, 128.9, 128.8, 127.8, 127.6; ESI-MS calcd for $C_{15}H_{12}NO$ $[M + H]^+$ 222.1, found 222.4.

Dibenzo[*b,f*]azocin-6(5H)-one (**2**, ref.1). Compound **1** (16 g, 72.3 mmol) was added to 250 mL polyphosphoric acid at 125 °C. After stirring for 60 min at 125 °C, the mixture was poured onto crushed ice (~700 mL), stirred for another 30 min, and filtered. The filter cake was washed with water, and dried under vacuum to provide crude dibenzo[*b*,*f*]azocin-6(5H)-one (**2**) as a grey powder in 73% yield (11.6 g, 52.4 mmol): ^{1}H NMR (400 MHz, $CDCl_3$) δ 7.94 (br. s., 1 H), 7.44-7.51 (m, 1 H), 7.22-7.33 (m, 2 H), 7.10-7.21 (m, 4 H), 7.03-7.08 (m, 1 H), 6.93-6.99 (m, 1 H), 6.81-6.89 (m, 1 H): ^{13}C NMR (100 MHz, $CDCl_3$) δ 173.8, 135.4, 135.2, 134.9, 134.2, 133.2, 129.9, 129.7, 129.3, 128.4, 128.4, 128.2, 127.7, 127.1, 126.4; ESI-MS calcd for $C_{15}H_{12}NO$ $[M + H]^+$ 222.1, found 222.3.

5,6-Dihydrodibenzo[*b,f*]azocine (**3**, ref.1). To a stirred suspension of lithium aluminum hydride (2.5 g, 67 mmol, 2 equiv.) in 150 mL anhydrous ether was added a suspension of **2** (7.4 g, 33 mmol, 1 equiv.) in anhydrous ether (20 mL) at 0 °C while the flask was purged with

nitrogen to prevent influx of air into a flask. The ice bath was removed and the mixture was heated under reflux for 12 h. The reaction was quenched at 0 °C by sequential addition of 2.5 mL of water, 2.5 mL of 15% NaOH, and 7.5 mL of water. The mixture was filtered, and the filter cake was washed with ether several times. The filter cake was dispersed in ether (100 mL), stirred for 10 min, and filtered. After separation of the organic layer of the filtrate, it was dried over anhydrous Na_2SO_4 and concentrated under reduced pressure. The residue was purified by flash column chromatography (hexane : EtOAc = 2:1) to provide **3** as a yellow-green powder in 58% yield (4.04 g, 19.5 mmol): ^{1}H NMR (400 MHz, $CDCl_3$) δ 7.28-7.22 (m, 1 H), 7.21-7.13 (m, 3 H), 6.96 (dd, *J* = 1.5, 7.3 Hz, 1 H), 6.91-6.83 (m, 1 H), 6.64-6.57 (m, 1 H), 6.53 (d, *J* = 13.2 Hz, 1 H), 6.46 (d, *J* = 8.1 Hz, 1 H), 6.35 (d, *J* = 12.5 Hz, 1 H), 4.57 (s, 2 H); ^{13}C NMR (100 MHz, $CDCl_3$) δ 147.3, 139.3, 138.3, 134.9, 132.7, 130.3, 129.0, 128.1, 127.8, 127.6, 127.5, 121.8, 118.1, 117.9, 49.6; ESI-MS calcd for $C_{15}H_{14}N$ $[M + H]^+$ 208.1, found 208.4.

Compound 4. To a stirred solution of **3** (0.75 g, 3.62 mmol, 1 equiv.) in DMF (50 mL) was sequentially added Fmoc-β-alanine (2.2 g, 7.2 mmol, 2 equiv.), HATU (2.4 g, 6.48 mmol, 1.8 equiv.), HOAt (0.98 g, 7.2 mmol, 2 equiv.) and DIEA (2.4 mL, 14 mmol, 4 equiv.). After stirring for 12 h, the mixture was diluted with EtOAc and washed with water three times. The organic layer was dried over anhydrous Na_2SO_4 and concentrated under reduced pressure. The residue was purified by flash column chromatography (hexane : EtOAc = 2:3) to provide **4** as a white solid in 71% yield (1.2 g, 2.56 mmol): ^{1}H NMR (400 MHz, $CDCl_3$) δ 7.79 (d, *J* = 7.3 Hz, 2 H), 7.61 (d, *J* = 7.3 Hz, 2 H), 7.47-7.39 (m, 2 H), 7.34 (t, *J* = 7.4 Hz, 2 H), 7.30-7.23 (m, 2 H), 7.22-7.14 (m, 3 H), 7.14-7.07 (m, 1 H), 6.73 (d, *J* = 13.1 Hz, 1 H), 6.54 (d, *J* = 12.8 Hz, 1 H), 5.57 (d, *J* = 15.3 Hz, 1 H), 5.52 (br. s., 1 H), 4.42-4.32 (m, 2 H), 4.29 (d, *J* = 15.3 Hz, 1 H), 4.24-4.16 (m, 1 H), 3.37 (br. s., 2 H), 2.34-2.21 (m, 1 H), 2.01 (td, *J* = 5.4, 16.0 Hz, 1 H); ^{13}C NMR (100 MHz, $CDCl_3$) δ 171.5, 156.4, 144.1, 141.4, 140.3, 136.4, 135.6, 134.5, 132.8, 132.3, 131.0, 130.1, 128.8, 128.4, 128.1, 127.8, 127.5, 127.2, 127.1, 125.3, 120.1, 66.7, 54.5, 47.3, 36.9, 35.0; ESI-MS calcd for $C_{33}H_{29}N_2O_3$ $[M + H]^+$ 501.2, found 501.2.

Compound 5. To a stirred solution of **4** (430 mg, 0.86 mmol, 1 equiv.) in CH_2Cl_2 (10 mL) was added pyridinium tribromide (330 mg, 1.0 mmol, 1.2 equiv.) at room temperature. After stirring for 8 h, the mixture was diluted with CH_2Cl_2 and washed with 5% aqueous HCl. The organic layer was dried over anhydrous Na_2SO_4 and concentrated under reduced pressure. The residue was passed through a short pad of silica gel (CH_2Cl_2) to give **5** as a yellowish solid in 94% yield (531 mg, 0.80 mmol): ^{1}H NMR (400 MHz, $CDCl_3$) δ 7.77 (d, *J* = 7.3 Hz, 2 H), 7.73 (dd, *J* = 0.7, 7.8 Hz, 1 H), 7.62 (br. s., 2 H), 7.44-7.37 (m, 2 H), 7.37-7.29 (m, 2 H), 7.24-7.17 (m, 2 H), 7.13 (dt, *J* = 1.3, 7.5 Hz, 1 H), 7.09-7.00 (m, 2 H), 6.96 (dd, *J* = 1.2, 7.6 Hz, 1 H), 6.90 (d, *J* = 7.3 Hz, 1 H), 5.91 (d, *J* = 9.8 Hz, 1 H), 5.85 (d, *J* = 14.7 Hz, 1 H), 5.67 (br. s., 1 H), 5.16 (d, *J* = 9.8 Hz, 1 H), 4.41-4.28 (m, 2 H), 4.26-4.16 (m, 2 H), 3.58-3.46 (m, 2 H), 2.61 (td, *J* = 5.4, 17.1 Hz, 1 H), 2.38 (td, *J* = 5.6, 17.0 Hz, 1 H); ^{13}C NMR (100 MHz, $CDCl_3$) δ 172.4, 156.5, 144.2, 144.1, 141.4, 138.2, 137.2, 136.7, 132.6, 131.0, 130.7, 130.6, 129.8, 129.7, 129.1,

128.7, 127.8, 127.2, 127.2, 125.3, 125.3, 120.1, 66.9, 60.1, 55.7, 52.5, 47.3, 37.0, 36.2; ESI-MS calcd for $C_{33}H_{29}Br_2N_2O_3$ $[M + H]^+$ 659.1, 661.1, 663.1 (1:2:1), found 659.2, 661.2, 663.3 (1:2:1).

Compound 6. To a stirred solution of **5** (400 mg, 0.61 mmol, 1 equiv.) in anhydrous THF (2 mL) was added potassium *t*-butoxide (204 mg, 1.8 mmol, 3 equiv.) in anhydrous THF (2 mL) at room temperature. After stirring for 1 h, the mixture was diluted with $CHCl_3$ (10 mL). To a stirred solution of the mixture was added succinic anhydride (55 mg, 0.55 mmol, 0.9 equiv.) at room temperature. After stirring for 4 h, the volatile material was removed under reduced pressure. The residue was purified by flash column chromatography (CH_2Cl_2 : MeOH = 20:1) to provide **6** as a brownish solid in 93% yield (215 mg, 0.57 mmol): ^{1}H NMR (400 MHz, $CDCl_3$) δ 7.64 (d, J = 7.3 Hz, 1 H), 7.43-7.22 (m, 7 H), 6.57 (br. s., 1 H), 5.12 (d, J = 14.2 Hz, 1 H), 3.69 (d, J = 13.9 Hz, 1 H), 3.40-3.29 (m, 1 H), 3.24-3.12 (m, 1 H), 2.66-2.53 (m, 2 H), 2.52-2.42 (m, 1 H), 2.39-2.28 (m, 2 H), 2.01-1.91 (m, 1 H): ^{13}C NMR (100 MHz, $CDCl_3$) δ 175.8, 172.5, 172.4, 150.9, 147.9, 132.2, 129.2, 128.9, 128.7, 128.5, 128.1, 127.4, 125.7, 123.0, 122.6, 114.9, 107.8, 55.8, 35.6, 34.6, 30.7, 30.0; ESI-MS calcd for $C_{22}H_{21}N_2O_4$ $[M + H]^+$ 377.2, found 377.6.

ADIBO-NHS (7). To a stirred solution of **6** (100 mg, 0.27 mmol, 1 equiv.) in CH_2Cl_2 (2 mL) were added EDC (61 mg, 0.32 mmol, 1.2 equiv.) and *N*-hydroxysuccinimide (NHS, 46 mg, 0.40 mmol, 1.5 equiv.) at room temperature. After stirred for 6 h, the mixture was washed with water three times and brine. The organic layer was dried over anhydrous Na_2SO_4 and concentrated under reduced pressure. The residue was purified by flash column chromatography (CH_2Cl_2 : MeOH = 98:2) to provide **7** as a white solid in 34% yield (44 mg, 0.1 mmol): ^{1}H NMR (400 MHz, $CDCl_3$) δ 7.67 (d, J = 7.5 Hz, 1 H), 7.41-7.26 (m, 7 H), 6.09 (t, J = 5.9 Hz, 1 H), 5.12 (d, J = 13.8 Hz, 1 H), 3.69 (d, J = 13.8 Hz, 1 H), 3.35 (dtd, J = 4.0, 6.9, 13.6 Hz, 1 H), 3.21 (dddd, J = 3.8, 5.7, 7.6, 13.5 Hz, 1 H), 2.91-2.85 (m, 2 H), 2.84-2.79 (m, 4 H), 2.45 (ddd, J = 3.9, 7.6, 16.6 Hz, 1 H), 2.41-2.34 (m, 2 H), 1.95 (ddd, J = 3.8, 7.3, 16.6 Hz, 1 H): ^{13}C NMR (100 MHz, $CDCl_3$) δ 172.3, 169.6, 169.1, 168.2, 151.1, 148.0, 132.2, 129.2, 128.7, 128.5, 128.5, 128.1, 127.4, 125.8, 123.0, 122.5, 114.8, 107.9, 55.6, 35.5, 34.7, 30.5, 26.7, 25.7; MALDI-TOF MS calcd for $C_{26}H_{24}N_3O_6$ $[M + H]^+$ 474.2, found 474.4.

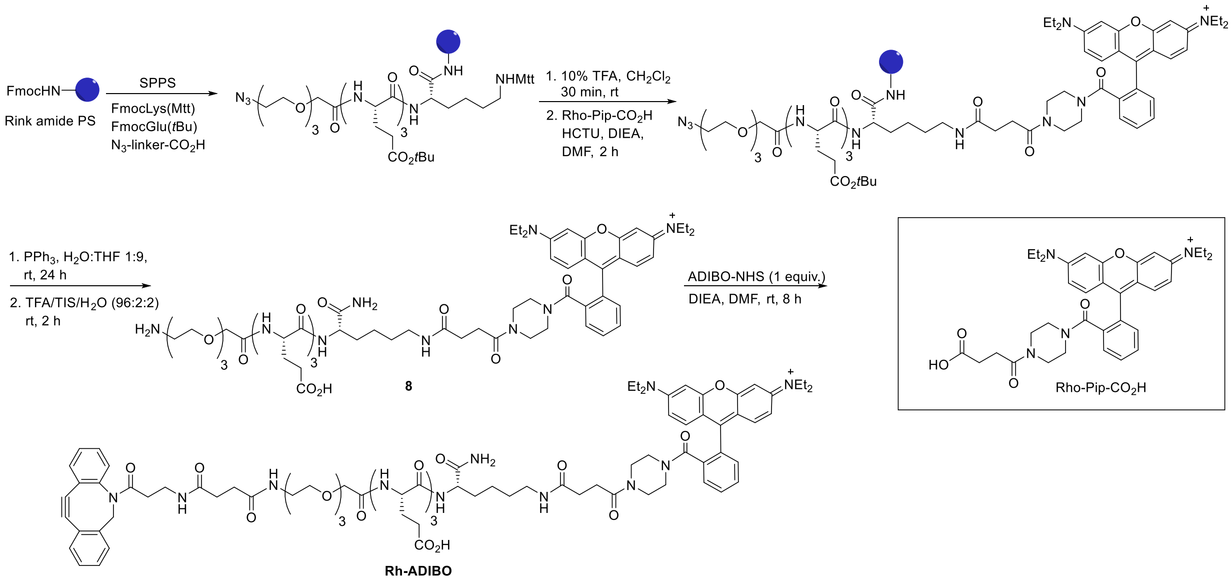

Compound 8. Compound **8** was synthesized by using the Fmoc/tBu strategy on Rink amide PS resin (0.9 mmol/g). Fmoc amino acids (3 equiv.) was manually coupled on resins (10 μmol) for 1 h in the presence of O-(6-chlorobenzotriazole-1-yl)-N,N,N',N'-tetramethyluronium hexafluorophosphate (HCTU, 2.8 equiv.) and N,N-diisopropylethylamine (DIEA, 6 equiv.). After coupling reaction, the Fmoc group was removed by treatment of 20% piperidine in DMF for 20 min twice, and the resin was washed thoroughly with DMF and CH_2Cl_2 several times. For coupling of Rho-Pip-CO_2H (ref. 2) to the resin, the Mtt protecting group was removed by treatment of the resin with 10% TFA in CH_2Cl_2 for 30 min and then the resultant resin was reacted with Rh-Pip-CO_2H (3 equiv.), DIEA (6 equiv.) and HCTU (2.8 equiv.) in DMF for 1 h. The terminal azide group on the resin was reduced by Staudinger reduction using 5% PPh_3 in THF and water (9:1) for 24 h. After washing the resin with DMF and CH_2Cl_2 several times, compound **8** was cleaved from the resin by treatment with TFA/triisopropylsilane/H_2O (96:2:2, v/v/v) for 2 h. The mixture was purified to provide **8** by preparative HPLC with gradient of 5-100% of acetonitrile (0.1% TFA) in H_2O (0.1% TFA) over 60 min: MALDI-TOF MS calcd for $C_{65}H_{92}N_{11}O_{18}^+$ $[M]^+$ 1314.7, found 1315.0.

Rh-ADIBO. To a solution of ADIBO-NHS (7 mg, 15 μmol, 1 equiv.) and **8** (20 mg, 15 μmol, 1 equiv.) in DMF (0.2 mL) at 0 °C was added DIEA (5 μL, 30 μmol, 2 equiv.). The mixture was shaken using an orbital shaker for 8 h at room temperature. The mixture was purified to provide **Rh-ADIBO** by preparative HPLC with gradient of 5-100% of acetonitrile (0.1% TFA) in H_2O (0.1% TFA) over 60 min: MALDI-TOF MS calcd for $C_{87}H_{110}N_{13}O_{21}^+$ $[M]^+$ 1672.8, found 1673.0.

<HPLC profiles of synthesized substances>

Analytic RP-HPLC (C18 column, 250 x 4.6 mm; pore size, 5 μM) with a gradient of 5-100% CH_3CN in water (0.1% TFA) over 45 min (a flow rate; 1 mL/min, detection at 350 nm).

Compound 8

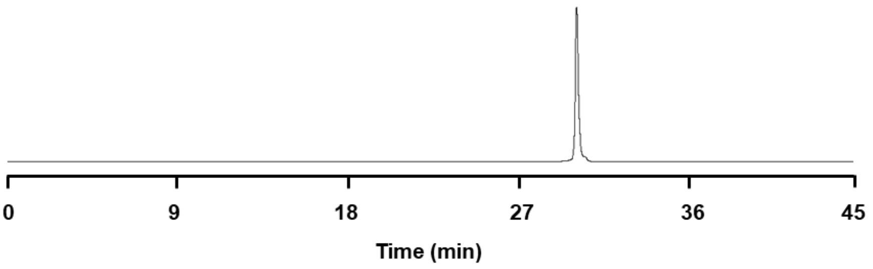

Rh-ADIBO

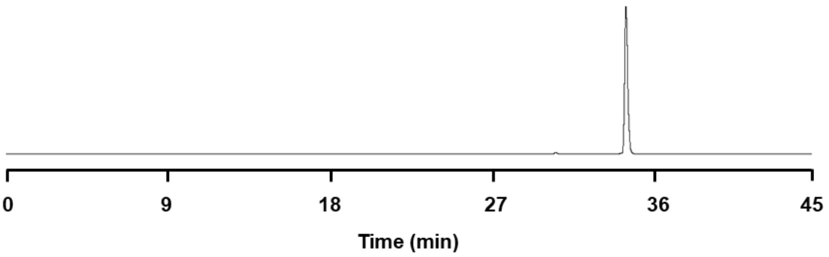

BIOLOGY

General. All commercially available chemicals and DNA oligomers were obtained from commercial sources and used without further purification. Fluorescence spectra were acquired on an Infinite® 200 PRO multimode microplate reader (Tecan). Live cell images were obtained by using confocal fluorescence microscopy (LSM 800, Zeiss).

Construction of plasmids. The EGFR gene (Sino Biological) was amplified by PCR using primers 1. The PCR product was digested with KpnI and XbaI and inserted into pcDNA3.1 (Addgene) to generate pcDNA-WT-EGFR. An amber codon (TAG) was introduced at 128, 274 or 380 position of EGFR in pcDNA-WT-EGFR by the overlap extension PCR using primers 2, 3 or 4, respectively, according to the manufacturer's protocol (Invitrogen, USA).

<primers used to subclone genes>

Primer list			
1. EGFR_WT	sense	5'-ATAGGTACCATGCGACCCTCCGGGACGG-3'	KpnI
	antisense	5'-TATTCTAGATCATGCTCCAATAAATTCACTGCTTTGTGGCG-3'	XbaI
2. EGFR_N128TAG	sense	5'-CTTATCTAACTATGATGCATAGAAAACCGGACTGAAGGAGC-3'	
	antisense	5'-GCTCCTTCAGTCCGGTTTTCTATGCATCATAGTTAGATAAG-3'	
3. EGFR_N274TAG	sense	5'-CTACAACCCCACCTAGTACCAGATGGATGTG-3'	
	antisense	5'-CACATCCATCTGGTACTAGGTGGGGTTGTAG-3'	
4. EGFR_F380TAG	sense	5'-GCATTTAGGGGTGACTAGTTCACACATACTCCTCCTC-3'	
	antisense	5'-GAGGAGGAGTATGTGTGAACTAGTCACCCCTAAATGC-3'	

Cell culture. HeLa cells were cultured in Dulbecco's Modified Eagle's Medium (DMEM) supplemented with 10% fetal bovine serum (FBS), 50 units/mL of penicillin, and 50 μg/mL of streptomycin at 37 ^{o}C in a humidified incubator with 5% CO_2.

Preparation of a stable cell line. HeLa cells in DMEM were plated in 6-well plates and supplemented with 2 μg of a plasmid containing a $tRNA_{CUA}^{EcLeu}$/AnapRS pair (Addgene) or a $tRNA_{CUA}^{Pyl}$/PylRS pair (Addgene) per well [plasmid (μg) : Viafect (μL, Promega) = 1:3]. After incubation for 24 h, cells were treated with trypsin-EDTA (0.25%, Sigma-Aldrich) and then transferred to plates containing DMEM supplemented with 600 μg/mL G418 and 10% FBS. After culturing for 10–20 days, the resistant cell colonies were transferred to a new culture flask using an aseptic pipette for further culture.

Incorporation of ANAP into EGFR in cells. HeLa cells stably transfected with pANAP were transfected with 1 μg of a plasmid containing the WT or mutant (N128TAG, N274TAG or F380TAG) EGFR gene per well [plasmid (μg) : Viafect (μL) = 1:3] in the presence of 50 μM ANAP for 24 h. Cells were briefly washed with Dulbecco's phosphate-buffered saline (DPBS) and then thoroughly washed with fresh culture media for 1 h three times (total 3 h washing).

Cell images were obtained by means of confocal fluorescence microscopy (λ_{ex} = 405 nm, collected at 410-530 nm for ANAP).

Construction of EGFR-AZDye 488 in cells. HeLa cells, stably transfected with a $tRNA_{CUA}^{Pyl}$/PylRS pair, were transfected with 1 μg of pcDNA-EGFR (WT, N128TAG, N274TAG or F380TAG) per well [plasmid (μg) : Viafect (μL) = 1:3] in the presence of 100 μM Lys(PA) for 48 h. After washing with DPBS, cells were subjected to copper-catalyzed azide-alkyne cycloaddition by addition of DPBS containing 10% DMEM, AZDye 488-N_3 (30 μM, Click Chemistry Tools), a premixed BTTAA-$CuSO_4$ complex ([BTTAA] = 200 μM, [$CuSO_4$] = 50 μM), and sodium ascorbate (500 μM). After 5 min incubation at room temperature, the solution was carefully removed by suction, and cells were washed with DPBS.

Real-time analysis of endocytosis of EGFR. HeLa cells expressing EGFR128-AZDye 488 in DMEM were treated with 50 ng/mL EGF (Prospec) or 1 μg/mL lectin (Vector Laboratories) over 30 min at 37 ^{o}C. For the competition study, HeLa cells expressing EGFR-AZDye 488 in DMEM were pre-treated with 1 μM erlotinib or EGFR antibody (5 μg/mL, Santa Cruz Biotechnology) over 1 h at 37 ^{o}C. The cells were the incubated with 50 ng/mL EGF over 30 min at 37 ^{o}C. After washing with DPBS, cells were exposed to 50 nM LysoTracker Deep Red (Invitrogen) for 30 min at 37 ^{o}C. After washing with DPBS, live cell images were obtained by using confocal fluorescence microscopy. The fluorescence of EGFR-AZDye 488 was detected in the 490-530 nm range with excitation at 488 nm, and that of LysoTracker Deep Red was monitored in the 645-700 nm range with excitation at 635 nm.

Quantitative analysis of fluorescence in cells. Fluorescence intensities in cells were quantified for each image using the mean region of interest (ROI) tool with the ZEN software. Specifically, a constant circular ROI was chosen to encompass the cell of interest, and the same ROI area size was used for background subtraction. The colocalization percentage of AZDye 488 with Lysotracker or Cy3 with Lysotracker was calculated by using the ImageJ software from Manders coefficient which was measured by using confocal fluorescence microscopy.

Labeling of cellular glycans with Rh-ADIBO. HeLa cells were incubated with 100 μM Ac_4ManNAz for 48 h. After washing with DPBS, cells were treated with 20 μM ADIBO-Rh in culture media for 5 min at 37 ^{o}C. After removing culture media, cells were washed with DPBS.

Dual-labeling of cells with Rh-ADIBO and AZDye 488-N_3. HeLa cells, stably transfected with a $tRNA_{CUA}^{Pyl}$/PylRS pair, were transfected with 1 μg of pcDNA-EGFR (WT, N128TAG, N274TAG or F380TAG) per well [plasmid (μg) : Viafect (μL) = 1:3] in the presence of 100 μM Lys(PA) and 100 μM Ac_4ManNAz for 48 h. After washing with DPBS, cells were incubated with 20 μM Rh-ADIBO in DMEM for 5 min at 37 ^{o}C. After removing culture media,

cells were washed with DPBS. Cells were then subjected to copper-catalyzed azide-alkyne cycloaddition by addition of DPBS containing 10% DMEM, AZDye 488-N_3 (30 μM), a premixed BTTAA-$CuSO_4$ complex ([BTTAA] = 200 μM, [$CuSO_4$] = 50 μM), and sodium ascorbate (500 μM). After 5 min incubation at room temperature, the solution was carefully removed by suction. After washing with DPBS, live cell images were obtained by means of confocal fluorescence microscopy. The fluorescence of EGFR-AZDye 488 was detected in live cells in the 490-530 nm range with excitation at 488 nm, and that of rhodamine-labeled glycans was monitored in the 570-630 nm range with excitation at 561 nm. The FRET ratio (I_{Rh}/I_{AZDye}) in live cells, where I_{Rh} and I_{AZDye} are the fluorescence intensities of rhodamine in the 570- 630 nm range and AZDye 488 in the 490-530 nm range with excitation at 488 nm, was determined.

In-gel fluorescence scanning. Treated cells were lysed with RIPA buffer (50 mM Tris-HCl, 150 mM NaCl, 1% NP-40, 1% CHAPS, 10% glycine, and one tablet of protease inhibitor cocktail (Roche), pH 7.4) for 10 min at 4 °C. Cell lysates were separated by 8% SDS-PAGE. The fluorescence of proteins on SDS-PAGE gels was visualized using a Typhoon FLA 9500 biomolecular imager (GE Healthcare) (AZDye 488 fluorescence; 473 nm excitation and BPB1 filter (collected at 520-540 nm), rhodamine fluorescence; 532 nm excitation and LPG filter (collected from 575 nm)).

Western blotting. Cell lysates were separated by 6 or 8% SDS-PAGE. The resolved proteins were transferred onto a polyvinylidene fluoride (PVDF) membrane (Millipore). The PVDF membrane was blocked with 5% (w/v) non-fat skim milk (VWR) in TBST (TBS buffer containing 0.5% Tween-20) for 1 h at room temperature to reduce nonspecific adsorption of antibodies. After washing with TBST, the membranes were incubated with diluted primary antibodies in TBST for 1 h at room temperature or overnight at 4 ^{o}C. After washing with TBST, the membranes were treated with secondary antibodies in TBST for 1 h at room temperature. The treated membranes were visualized by using a Miracle-Star Western Blot Detection System (Intron Biotechnology Inc., South Korea). The signals were detected by G:BOX Chemi XT4 (ChemiFluorescent & Chemiluminescent Imaging System). Mouse EGFR (1:1000, Santa Cruz Biotechnology), mouse p-Tyr EGFR (1:1000, Santa Cruz Biotechnology) and mouse β-actin (1:1000, Santa Cruz Biotechnology) were used as primary antibodies. Horseradish peroxidase (HRP)-conjugated goat anti-mouse IgG (1:1000, Santa Cruz Biotechnology) were used as the secondary antibody.

Lectin blotting. Cell lysates were separated by 8% SDS-PAGE. The resolved proteins were transferred onto a polyvinylidene fluoride (PVDF) membrane (Millipore). The PVDF membrane was blocked with 5% (w/v) non-fat skim milk (VWR) in TBST (TBS buffer containing 0.5% Tween-20) for 1 h at room temperature and then incubated with biotinylated SNL (2 μg/mL in TBST, Vector Laboratories) or AAL (2 μg/mL in TBST, Vector Laboratories) for 1 h at room temperature. After washing with TBST, the membranes were incubated with 100 ng/mL streptavidin-HRP in TBST for 1 h at room temperature. After washing, the blots

were developed using a Miracle-Star Western Blot Detection System (Intron Biotechnology Inc., South Korea). The signals were detected using G:BOX Chemi XT4 (ChemiFluorescent & Chemiluminescent Imaging System).

Treatment of cells with glycosidases. HeLa cells were treated with α1-2,3,4,6 fucosidase (0.2 U/mL, NEB) or α2-3,6,8,9 sialidase (0.04 U/mL, Roche) for 1 h at 37 ^{o}C. After washing with DPBS, cells were lysed with RIPA buffer. Cell lysates were separated with 6 or 8% SDS-PAGE and analyzed by Western blotting and lectin blotting.

EGFR dimerization assay. HeLa cells expressing EGFR-AZDye 488 in DMEM were treated with 50 ng/mL EGF (Prospec) or 1 μg/mL lectin (Vector Laboratories) over 30 min at 37 ^{o}C. After washing with ice-cold DPBS while maintaining cells on ice, cells were treated with 3 mM suberic acid bis(3-sulfo-N-hydroxysuccinimide ester) sodium salt (Sigma-Aldrich) in ice-cold DPBS for 20 min on ice. After washing with cold DPBS, cells were lysed with RIPA buffer. Proteins were separated by 6% SDS-PAGE under non-reducing conditions and analyzed by Western blotting with EGFR antibody.

Immunoprecipitation assays. HeLa cells containing a $tRNA_{CUA}^{Pyl}$/PylRS pair were transfected with an EGFR-N128TAG gene in the presence of 100 μM Lys(PA) for 48 h at 37 ^{o}C. Cells were incubated with α1-2,3,4,6 fucosidase (0.2 U/mL) or α2-3,6,8,9 sialidase (0.04 U/mL) for 1 h at 37 ^{o}C. After washing with DPBS, cells were lysed with RIPA buffer for 10 min at 4 °C. After centrifugation at 15,000 rpm for 10 min at 4 ^{o}C, cell lysates were pre-incubated with 20 μL of Protein G Plus-agarose (Santa Cruz Biotechnology) in RIPA buffer for 30 min at 4 ^{o}C. The pre-cleared cell lysates were incubated with EGFR antibody for 2 h at 4 ^{o}C and then with Protein G Plus-agarose on the rocker at 4 ^{o}C overnight. Immunoprecipitates were collected by centrifugation at 2,500 rpm for 5 min at 4 ^{o}C. After washing with RIPA buffer, the supernatant was discarded, and pellets were re-suspended with 40 μL of 2X electrophoresis sample buffer. Amounts of immunoprecipitates were analyzed by Western blotting or lectin blotting.

Measurement of cell death. Cell viability was assessed by using an MTT ((3-(4,5-dimethylthiazol-2-yl)-2,5-diphenyltetrazolium bromide) assay. HeLa cells (5 x 10^3 cells/100 μL), stably transfected with a $tRNA_{CUA}^{Pyl}$/PylRS pair, were plated in triplicate in 96-well plates for 24 h and then incubated with $Ac_4ManNAz$, Lys(PA) or both in DMEM for 48 h. MTT (20 μL of 5 mg/mL solution) was added to culture media in each well, and the mixture was then incubated for 2 h. After removing the culture media containing MTT, 100 μL of DMSO was added and incubated for 30 min for color development. Absorbance at 590 nm was measured using an Infinite® 200 PRO multimode microplate reader (Tecan, Switzerland).

Preparation of Cy3-labeled lectin. Cy3-NHS ester (GE Healthcare) in DMSO was added to each lectin (1 mg) in 10 mM $NaHCO_3$ buffer (pH 8.0) according to the manufacturer's protocol. The mixture was vortexed for 1 h at room temperature. Low-molecular mass materials were removed by using a PD-10 column (GE Healthcare) and diluted with PBS (pH7.6) containing 0.1% (w/v) sodium azide.

Supplementary Reference

1. A. Zhou, J. Du, M. Jiao, D. Xie, Q. Wang, L. Xue, C. Ju, Z. Hua and C. Zhang, *J. Control. Release*, 2019, **304**, 111-124.

2. Y. Yasueda, T. Tamura, A. Fujisawa, K. Kuwata, S. Tsukiji, S. Kiyonaka and I. Hamachi,. *Am. Chem. Soc.*, 2016, **138**, 7592-7602.

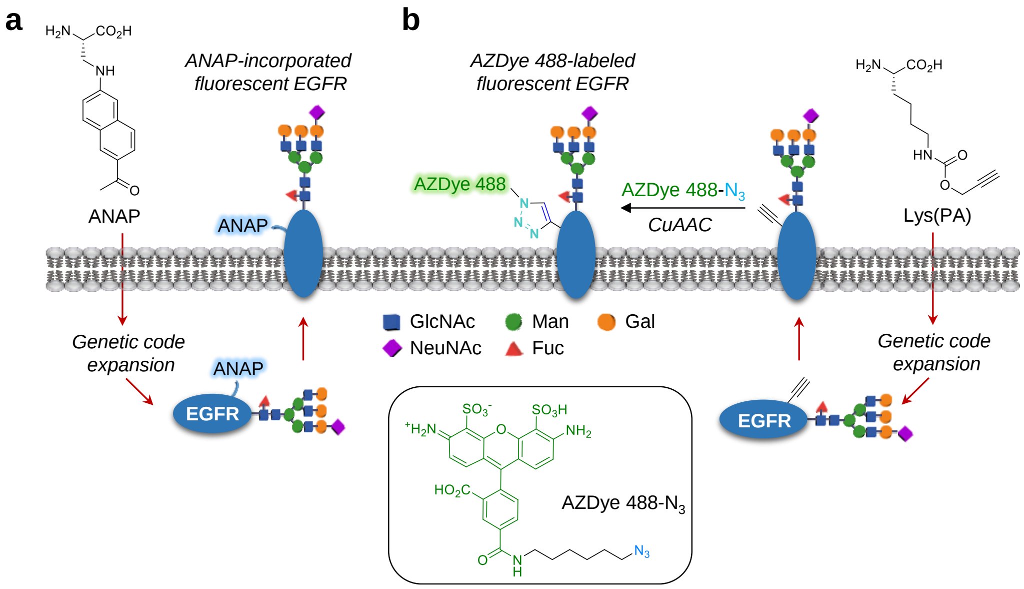

Figure S1. Construction of fluorescent EGFR on the cell surface. (a) ANAP is incorporated into EGFR using the genetic code expansion technique. (b) Lys(PA) is inserted into EGFR using the genetic code expansion technique. Subsequently, the alkyne group of Lys(PA) in EGFR is labeled with AZDye 488-N_3 via copper-catalyzed azide-alkyne cycloaddition (CuAAC).

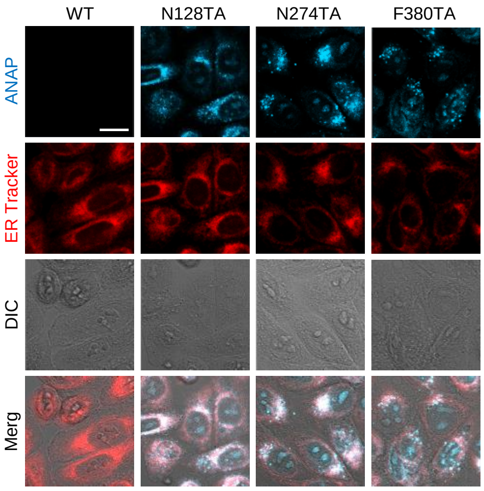

Figure S2. HeLa cells, stably transfected with pANAP containing a $tRNA_{CUA}^{EcLeu}$/AnapRS pair, were transfected with a plasmid containing a WT or mutant EGFR gene in the presence of 50 μM ANAP for 24 h. After cells were thoroughly washed with fresh media, cells were treated with ER-tracker Red (λ_{ex} = 561 nm, detected in the 570-630 nm). Live cell images were obtained by using confocal fluorescence microscopy (scale bar: 10 μm). The ANAP fluorescence was detected in live cells in the 410–530 nm range with excitation at 405 nm. The results showed that ANAP fluorescence is greatly colocalized with an ER-tracker signal. Thus, it is likely that misfolded ANAP-incorporated EGFR is somehow trapped into ER.

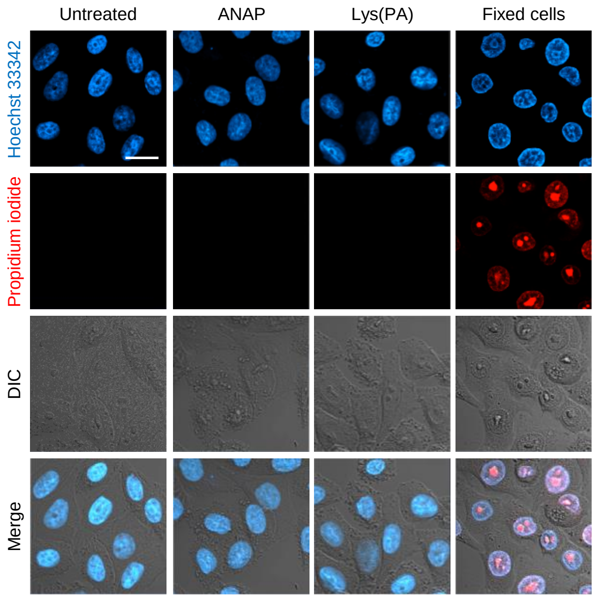

Figure S3. HeLa cells were incubated with 100 μM Lys(PA) for 48 h or 50 μM ANAP for 24 h. Cells were stained with Hoechst 33342 and propidium iodide (PI). Fixed cells as a control were stained with Hoechst 33342 and propidium iodide (PI). Cell images were obtained by using confocal fluorescence microscopy (scale bars: 20 μm). The PI fluorescence was detected in cells in the 570-700 nm range with excitation at 561 nm. The results showed that the PI fluorescence is not detected in treated cells unlike fixed cells as a control. The findings indicate that treatment of cells with 50 μM ANAP or 200 μM Lys(PA) does not perturb cell membranes.

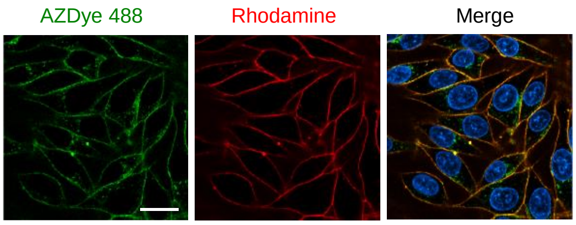

Figure S4. HeLa cells, stably transfected with a plasmid containing a $tRNA_{CUA}^{Pyl}$/PylRS pair, were transfected with an EGFR-N128TAG gene in the presence of 100 μM Lys(PA). Cells were then subjected to CuAAC with 30 μM AZDye 488-N_3 followed by exposure to 10 μM Rh-DHPE (rhodamine-labeled 1,2-dihexadecanoyl-sn-glycero-3-phosphoethanolamine) for 30 min. Live cell images were obtained by using confocal fluorescence microscopy (scale bar: 20 μm). Right: merged cell images of Hoechst 33342 (detected in the 410-460 nm range with excitation at 405 nm), AZDye 488 (detected in the 490-530 nm range with excitation at 488 nm) and rhodamine fluorescence (detected in the 570-630 nm range with excitation at 561 nm).

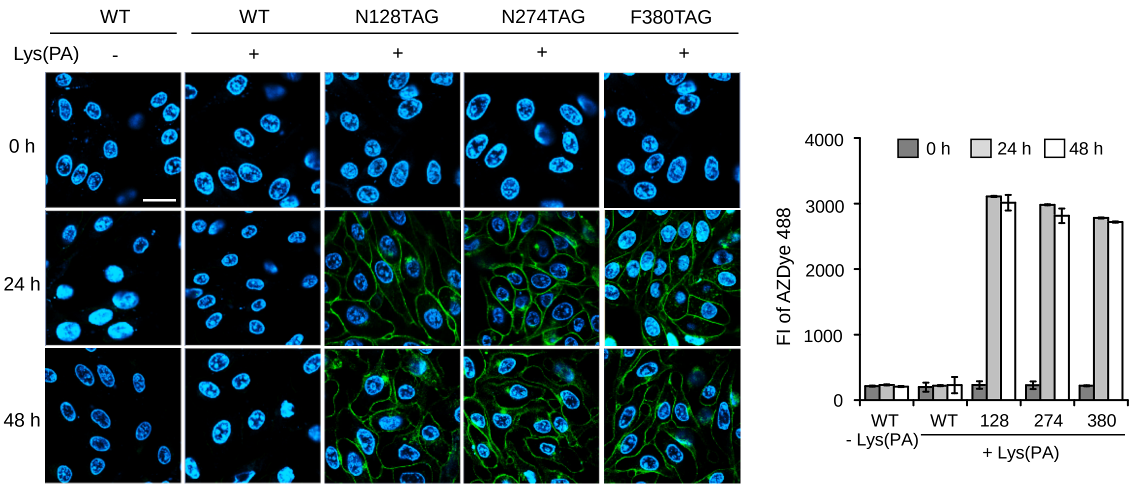

Figure S5. Time-dependent incorporation of Lys(PA) into EGFR. HeLa cells, stably transfected with a plasmid containing a $tRNA_{CUA}^{Pyl}$/PylRS pair, were transfected with an indicated EGFR gene in the presence of 100 μM Lys(PA) during indicated time periods. Cells were then subjected to CuAAC with 30 μM AZDye 488-N_3. The nucleus was stained with Hoechst 33342. Live cell images were obtained by using confocal fluorescence microscopy (scale bar: 20 μm). Shown are merged cell images of Hoechst 33342 and AZDye 488 fluorescence. Graph shows the fluorescence intensity (FI) of AZDye 488 in cells (mean ± s.d., n = 3).

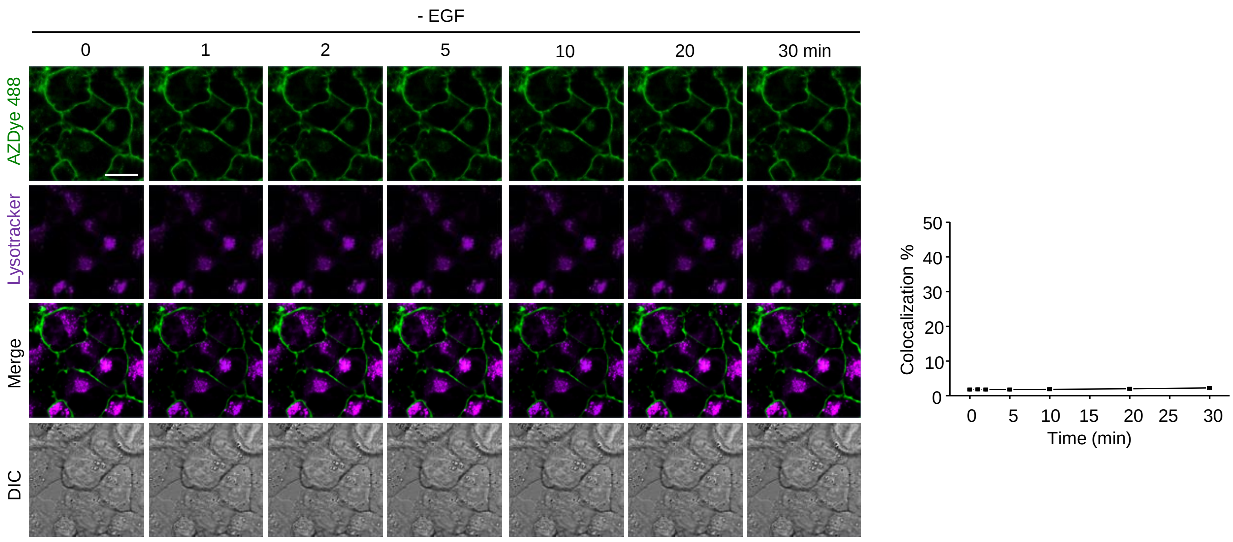

Figure S6. Time-dependent endocytosis of EGFR. HeLa cells containing a $tRNA_{CUA}^{Pyl}$/PylRS pair were transfected with an EGFR-N128TAG gene in the presence of 100 μM Lys(PA) for 48 h. Cells were treated with 30 μM AZDye 488-N_3 followed by Lysotracker Deep Red (λ_{ex} = 635 nm, detection in the 645-700 nm range). Live cell images were collected at selected time by using confocal fluorescence microscopy (scale bar: 20 μm). Graph shows the percentage of time-dependent colocalization of AZDye 488 with Lysotracker fluorescence, quantified by fluorescence image analysis (mean ± s.d., n = 3).

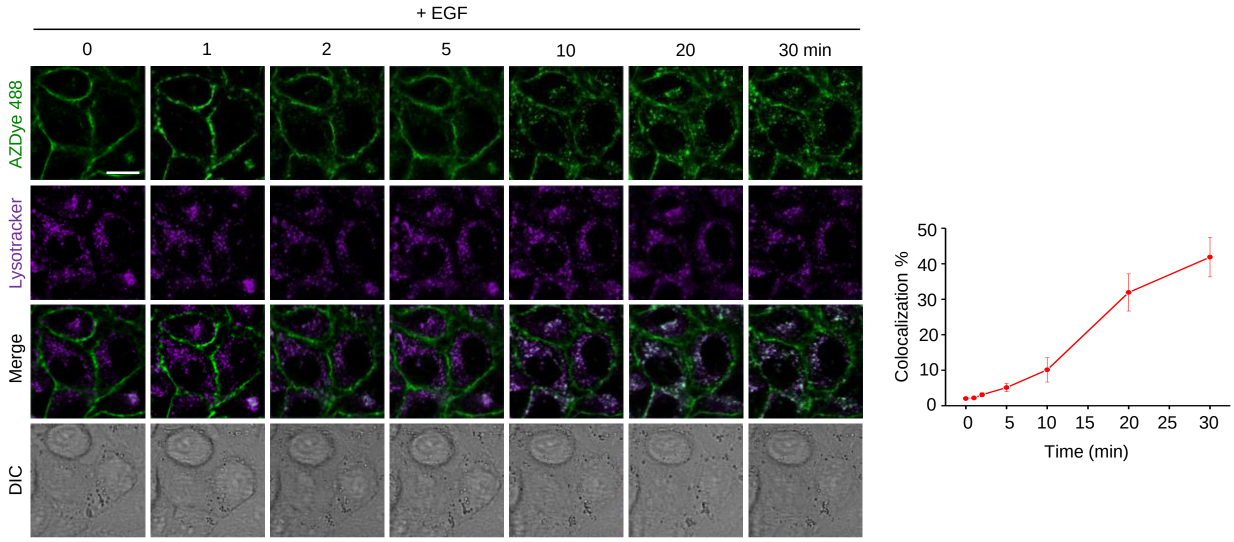

Figure S7. Time-dependent endocytosis of EGFR. HeLa cells containing a $tRNA_{CUA}^{Pyl}$/PylRS pair were transfected with an EGFR-N128TAG gene in the presence of 100 μM Lys(PA) for 48 h. Cells were treated with 30 μM AZDye 488-N_3 followed by exposure to Lysotracker Deep Red. After cells were incubated with 50 ng/mL EGF, live cell images were collected at selected time post incubation of EGF by using confocal fluorescence microscopy (scale bar: 20 μm). Graph shows the percentage of time-dependent colocalization of AZDye 488 with Lysotracker fluorescence (mean ± s.d., n = 3).

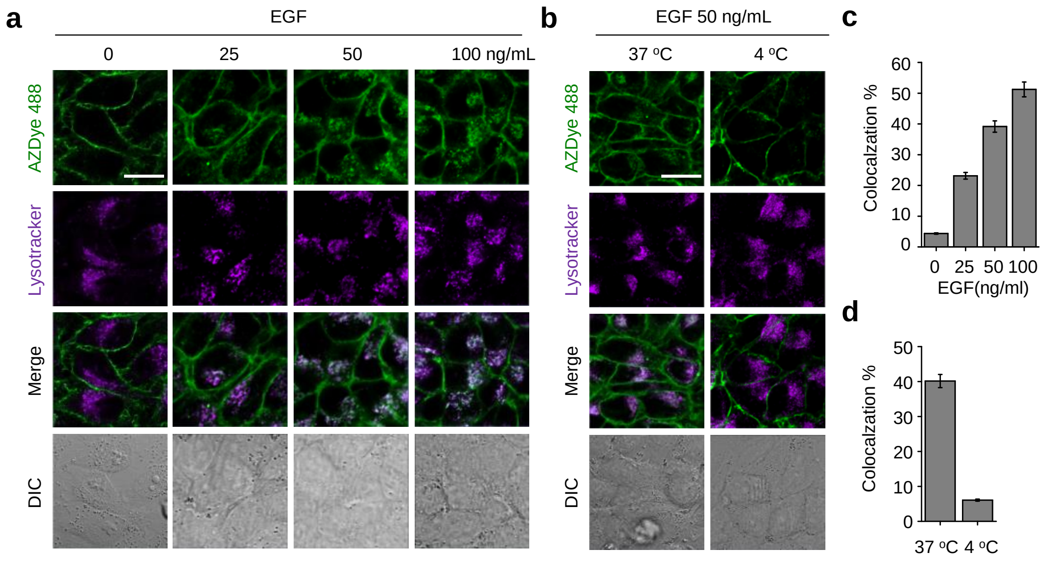

Figure S8. HeLa cells containing a $tRNA_{CUA}^{Pyl}$/PylRS pair were transfected with an EGFR-N128TAG gene in the presence of 100 μM Lys(PA) for 48 h. Cells were treated with 30 μM AZDye 488-N_3 followed by exposure to Lysotracker Deep Red (λ_{ex} = 635 nm, detection in the 645-700 nm range). Cells were then incubated for 30 min with (a) 0 - 100 ng/mL EGF at 37 °C and (b) 50 ng/mL EGF at 37 °C or 4 °C. Live cell images were obtained by using confocal fluorescence microscopy (scale bar: 20 μm). (c) and (d) Percentage of colocalization of Lysotracker with AZDye 488 fluorescence in (a) and (b), quantified by fluorescence image analysis (mean ± s.d., n = 3).

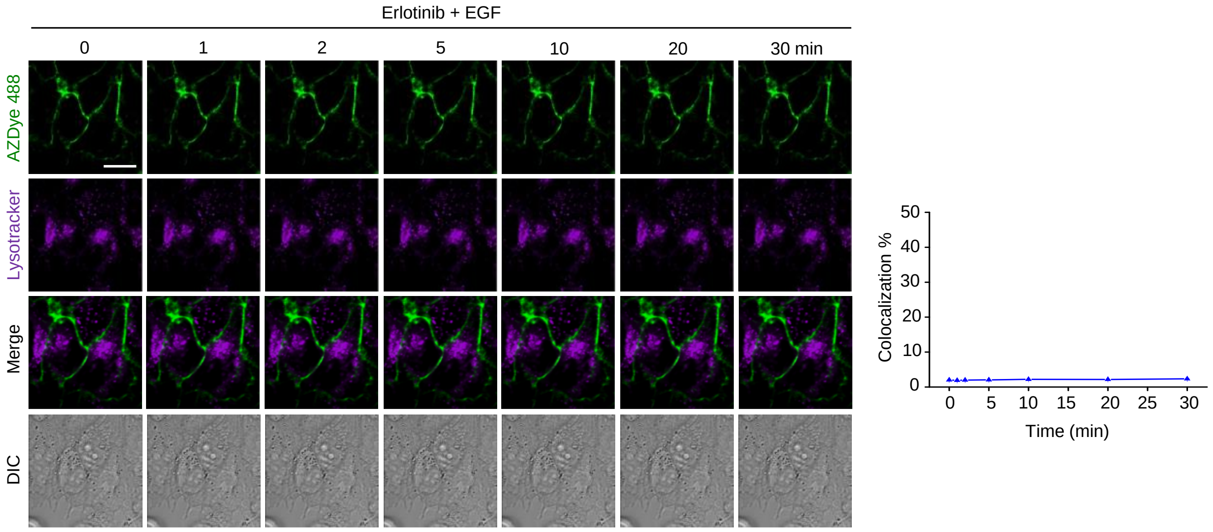

Figure S9. Time-dependent endocytosis analysis of EGFR. The fluorescently labeled cells were treated with Lysotracker Deep Red and then incubated with 50 ng/mL EGF in the presence of 1 μM erlotinib. Live cell images were collected at selected time post incubation of EGF by using confocal fluorescence microscopy (scale bar = 20 μm). Graph shows the percentage of time-dependent colocalization of AZDye 488 with Lysotracker fluorescence (mean ± s.d., n = 3).

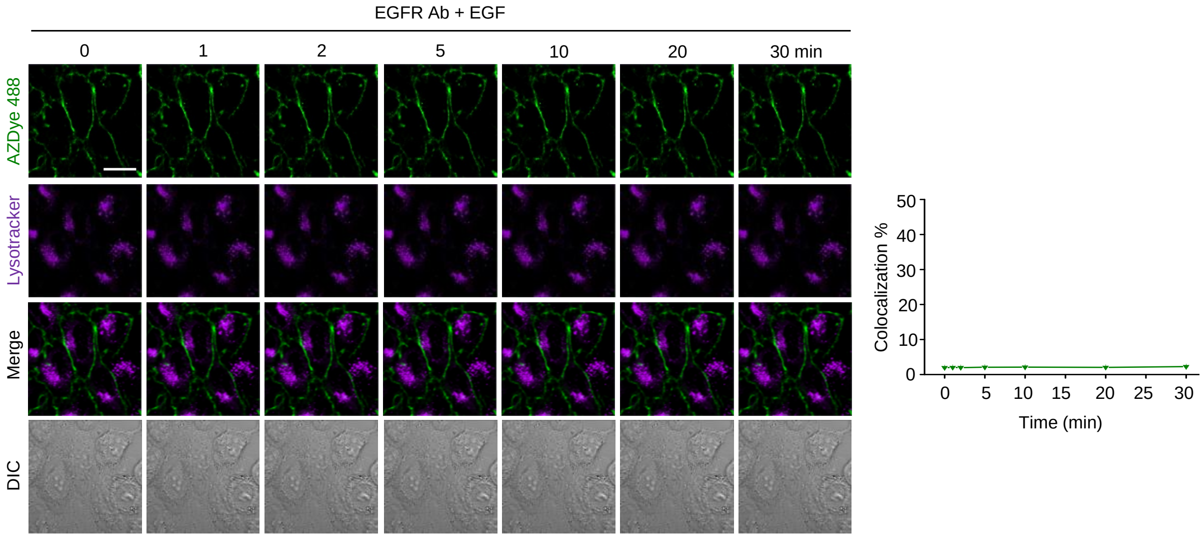

Figure S10. Time-dependent endocytosis analysis of EGFR. The fluorescently labeled cells were treated with Lysotracker Deep Red and then incubated with 50 ng/mL EGF in the presence of EGFR antibody. Live cell images were collected at selected time post incubation of EGF by using confocal fluorescence microscopy (scale bar = 20 μm). Graph shows the percentage of time-dependent colocalization of AZDye 488 with Lysotracker fluorescence (mean ± s.d., n = 3).

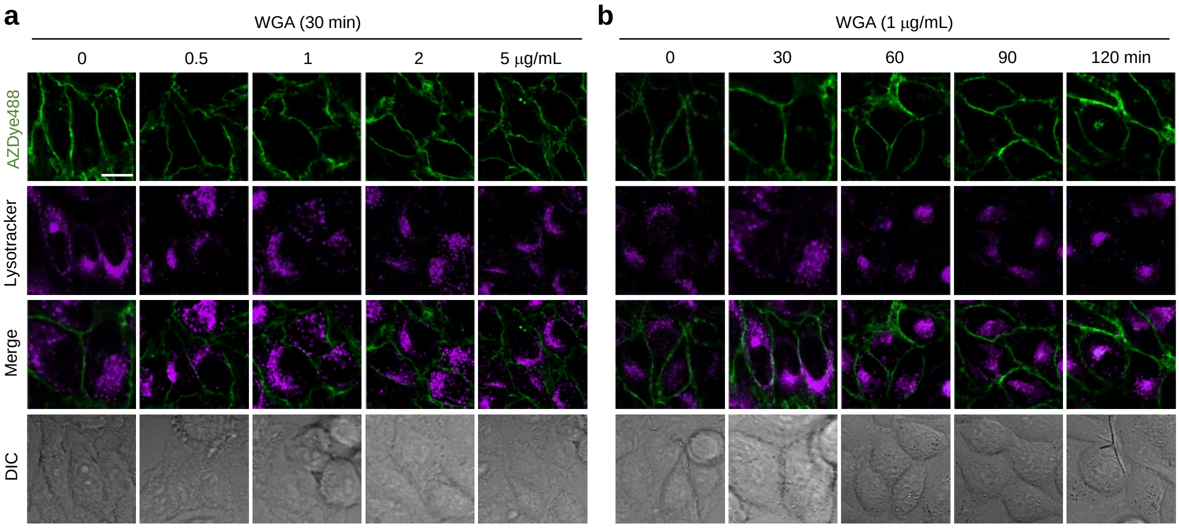

Figure S11. HeLa cells containing a $tRNA_{CUA}^{Pyl}$/PylRS pair were transfected with an EGFR-N128TAG gene in the presence of 100 μM Lys(PA) for 48 h. Cells were then subjected to CuAAC with 30 μM AZDye 488-N_3. The fluorescently labeled cells were treated with Lysotracker Deep Red followed by exposure to (a) indicated concentrations of WGA for 30 min and (b) 1 μg/mL WGA for indicated times. Live cell images were obtained by using confocal fluorescence microscopy (scale bar: 20 μm).

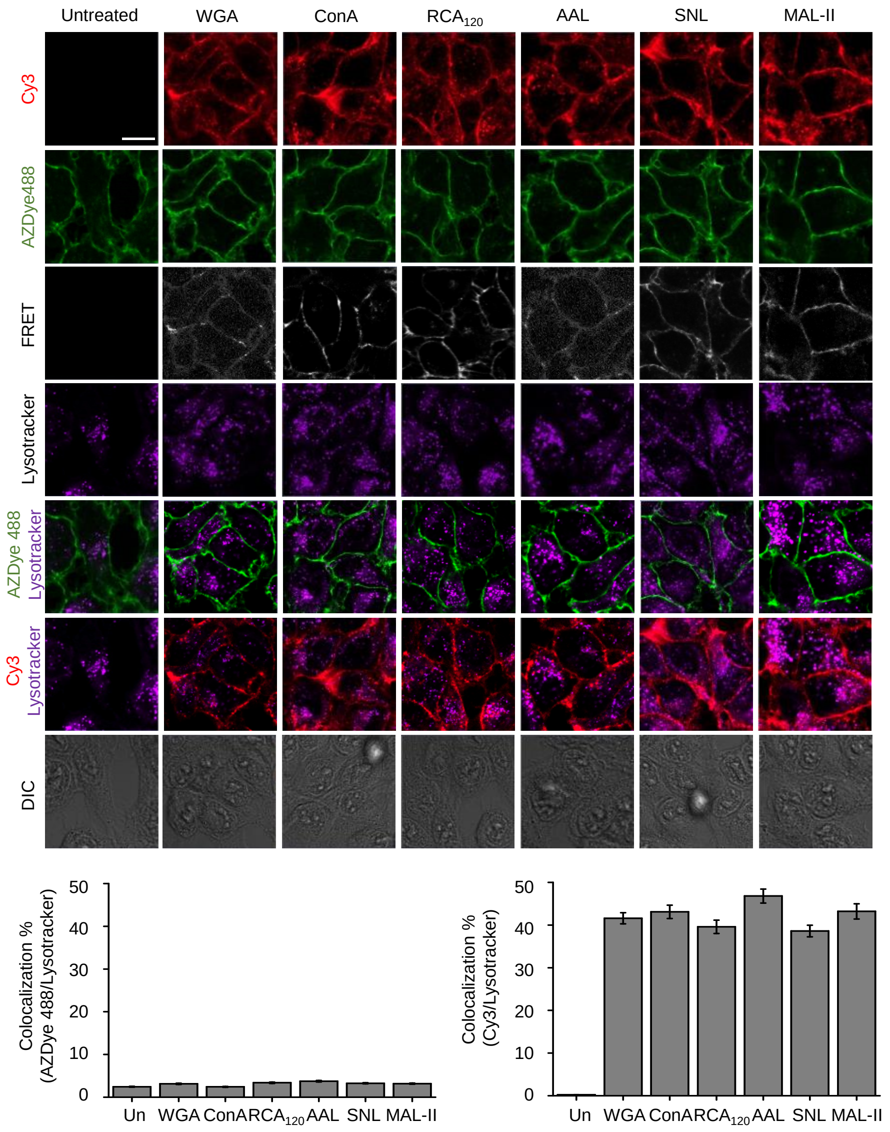

Figure S12. HeLa cells containing a tRNA$_{CUA}^{Pyl}$/PylRS pair were transfected with an EGFR-N128TAG gene in the presence of 100 μM Lys(PA) for 48 h. Cells were then subjected to CuAAC with 30 μM AZDye 488-N_3. The fluorescently labeled cells were treated with Lysotracker Deep Red followed by 30 min exposure to 1 μg/mL of each Cy3-labeled lectin (λ_{ex} = 561 nm, detected in the 570-630 nm range). Live cell images were obtained by using confocal fluorescence microscopy (scale bar = 20 μm). Graphs show the percentage of colocalization of (left) AZDye 488 with Lysotracker fluorescence and (right) Cy3 with Lysotracker fluorescence (mean ± s.d., n = 3).

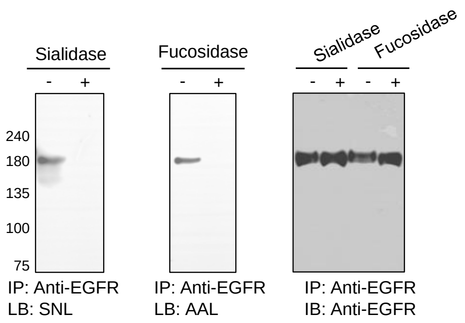

Figure S13. HeLa cells containing a $tRNA_{CUA}^{Pyl}$/PylRS pair were transfected with an EGFR-N128TAG gene in the presence of 100 μM Lys(PA) for 48 h. Cells were incubated with either fucosidase or sialidase for 1 h and immunoprecipitated with EGFR antibody followed by lectin or Western blotting.

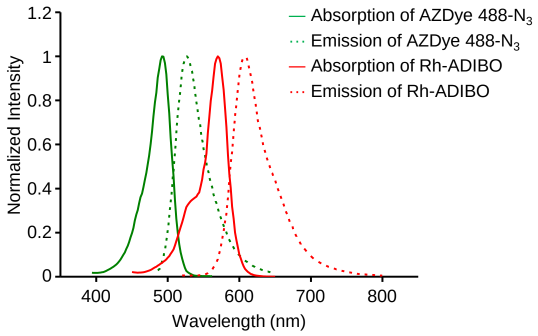

Figure S14. Normalized absorption and emission spectra of AZDye 488-N_3 and Rh-ADIBO.

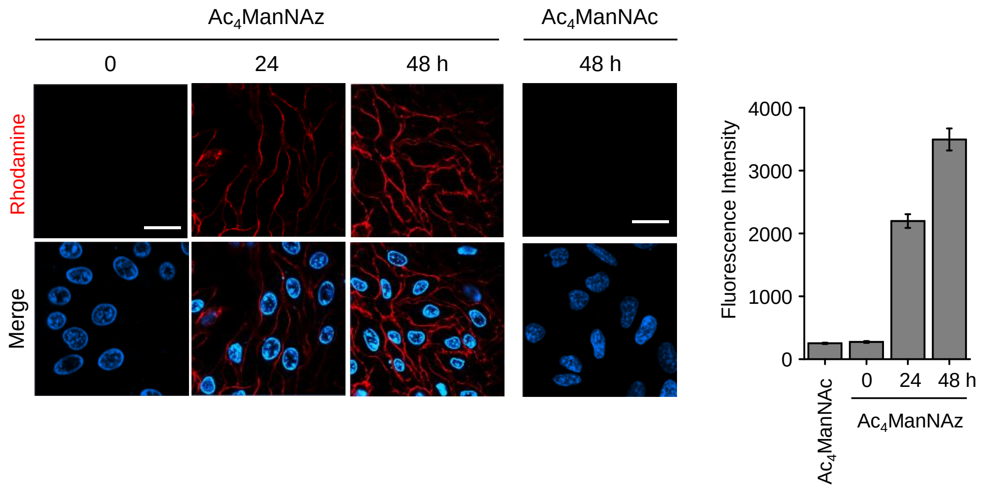

Figure S15. Time-dependent metabolic incorporation of $Ac_4ManNAz$ into cellular glycans. HeLa cells were incubated with 100 μM $Ac_4ManNAz$ during indicated time periods and then treated with 20 μM Rh-ADIBO. As a control, HeLa cells were also incubated with 100 μM $Ac_4ManNAc$ for 48 h followed by exposure to 20 μM Rh-ADIBO. Live cell images were obtained by using confocal fluorescence microscopy (scale bar: 20 μm). Bottom images: merged cell images of Hoechst 33342 and rhodamine fluorescence. Graph shows the fluorescence intensity of rhodamine in cells (mean ± s.d., n = 3).

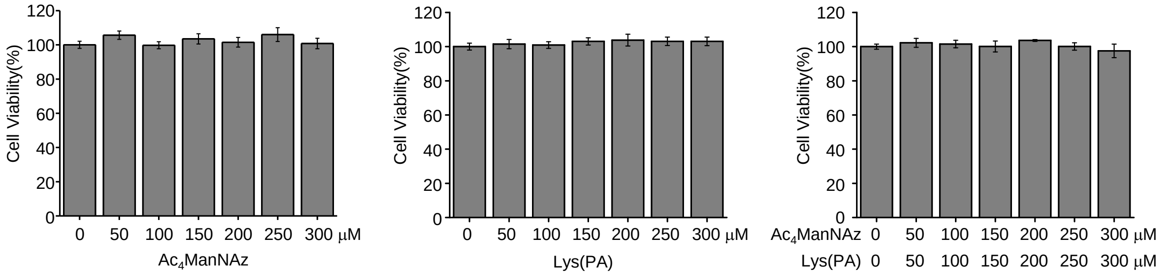

Figure S16. HeLa cells, stably transfected with a plasmid containing a $tRNA_{CUA}^{Pyl}$/PylRS pair, were treated with various concentrations of indicated substances for 48 h. Cell viabilities were measured by means of an MTT assay (mean ± s.d., n=3).

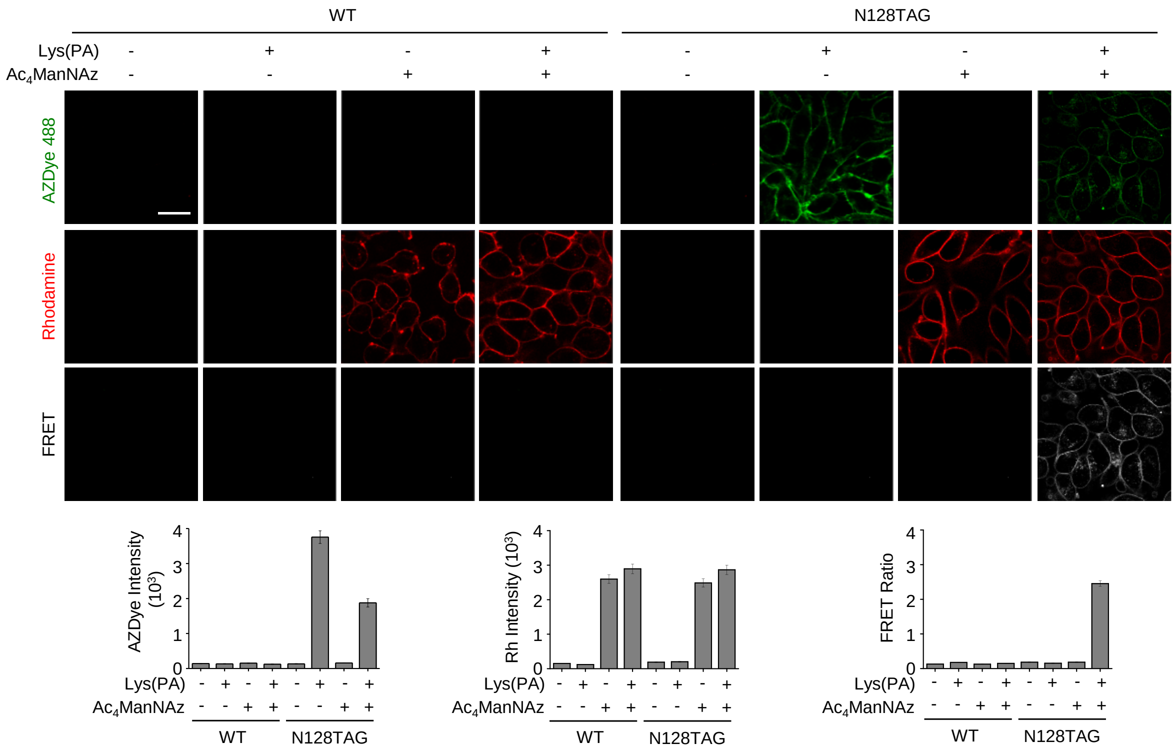

Figure S17. FRET-based detection of EGFR-specific glycosylation. HeLa cells, stably transfected with a plasmid containing a $tRNA_{CUA}^{Pyl}$/PylRS pair, were transfected with an EGFR gene (WT or N128TAG) in the presence or absence of 100 μM Lys(PA) or/and 100 μM $Ac_4ManNAz$ for 48 h. Cells were then treated sequentially with 20 μM Rh-ADIBO and 30 μM AZDye 488-N_3. Live cell images were obtained by using confocal fluorescence microscopy (scale bar: 20 μm). Graphs show fluorescence intensities of AZDye 488 (λ_{ex} = 488 nm) and rhodamine (λ_{ex} = 561 nm) as well as a FRET ratio (I_{Rh}/I_{AZ488}, λ_{ex} = 488 nm) in cells (mean ± s.d., n = 3).

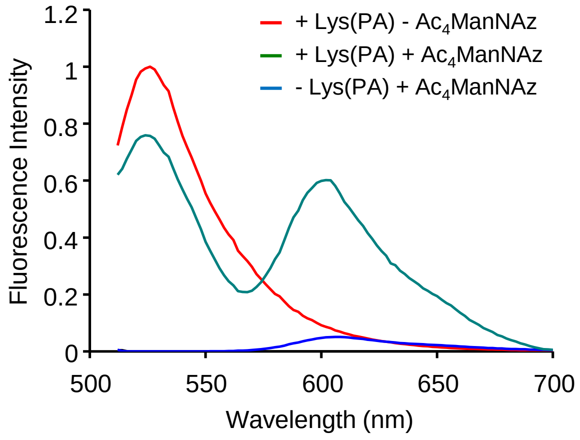

Figure S18. Fluorescence spectra of cells transfected with an EGFR-N128TAG gene in the presence or absence of Lys(PA) and Ac_4ManNAz followed by labeling with Rh-ADIBO and AZDye 488-N_3(λ_{ex} = 488 nm). The emission maximum around 600 nm in green represents the FRET signal.

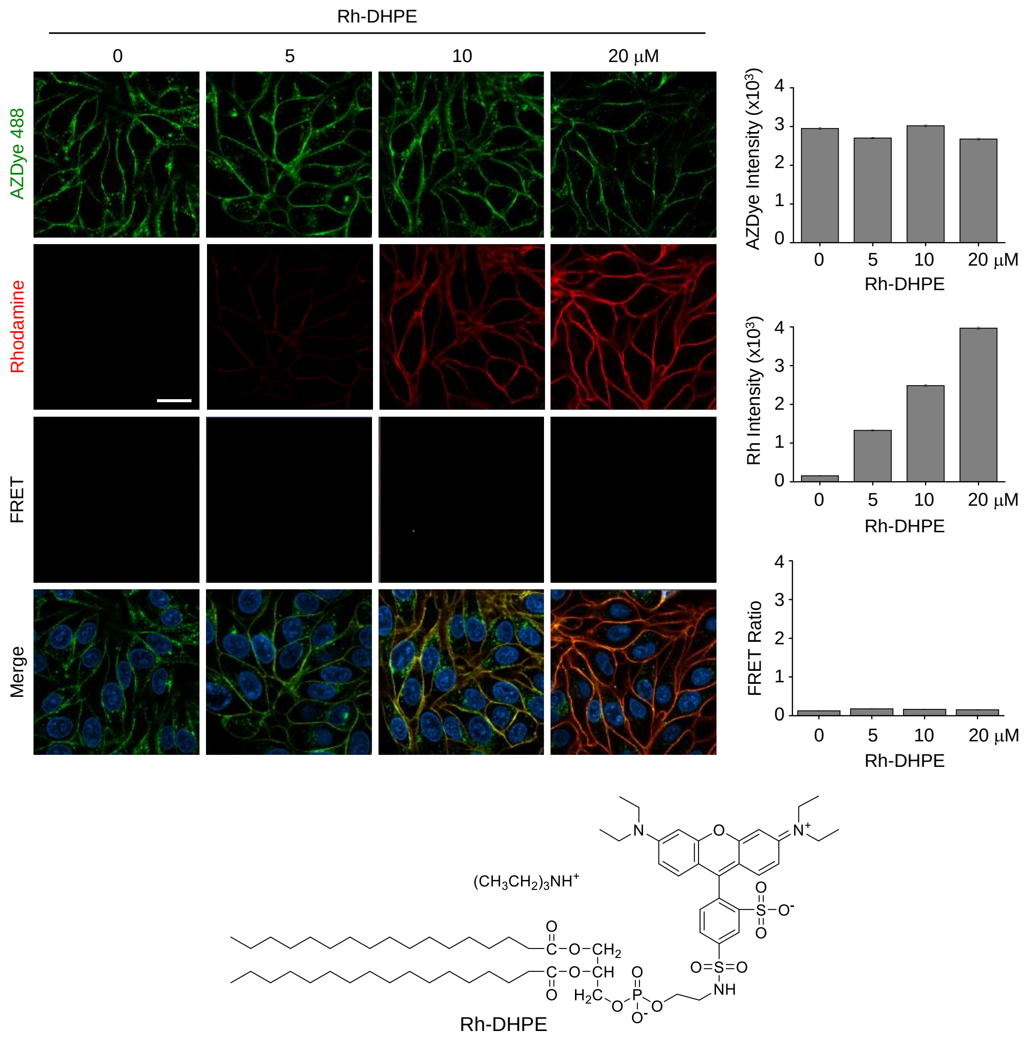

Figure S19. HeLa cells containing a $tRNA_{CUA}^{Pyl}$/PylRS pair were transfected with an EGFR-N128TAG gene in the presence of 100 μM Lys(PA) for 48 h. Cells were then treated with various concentrations of Rh-DHPE for 30 min followed by exposure to 30 μM AZDye 488-N_3. Live cell images were obtained by using confocal fluorescence microscopy (scale bar: 20 μm). Graphs show fluorescence intensities of AZDye 488 (λ_{ex} = 488 nm) and rhodamine (λ_{ex} = 561 nm) as well as a FRET ratio (I_{Rh}/I_{AZDye}, λ_{ex} = 488 nm) in cells (mean ± s.d., n = 3).

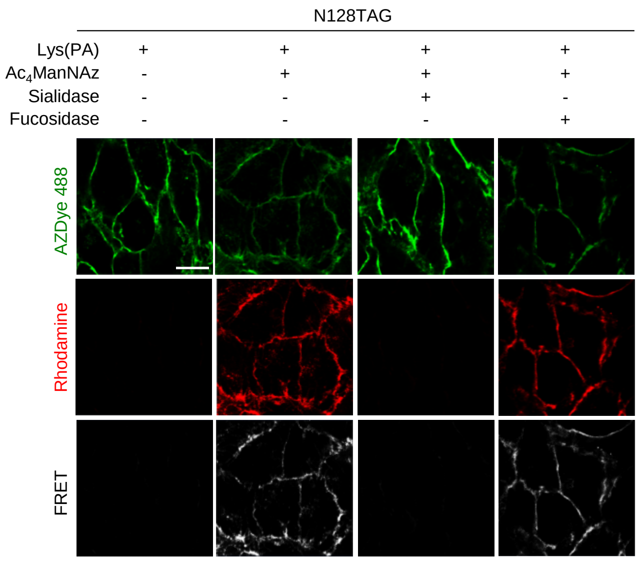

Figure S20. HeLa cells, stably transfected with a plasmid containing a $tRNA_{CUA}^{Pyl}$/PylRS pair, were transfected with an EGFR-N128TAG gene in the presence or absence of 100 μM Lys(PA) and 100 μM Ac_4ManNAz for 48 h. After treatment with sialidase or fucosidase to remove cell-surface sialic acids or fucoses, cells were incubated with 20 μM Rh-ADIBO followed by exposure to 30 μM AZDye 488-N_3. Live cell images were obtained by using confocal fluorescence microscopy (scale bars: 20 μm).

<NMR spectra>

Compound 1: $CDCl_3$, 400 MHz ^{1}H NMR, 100 MHz ^{13}C NMR

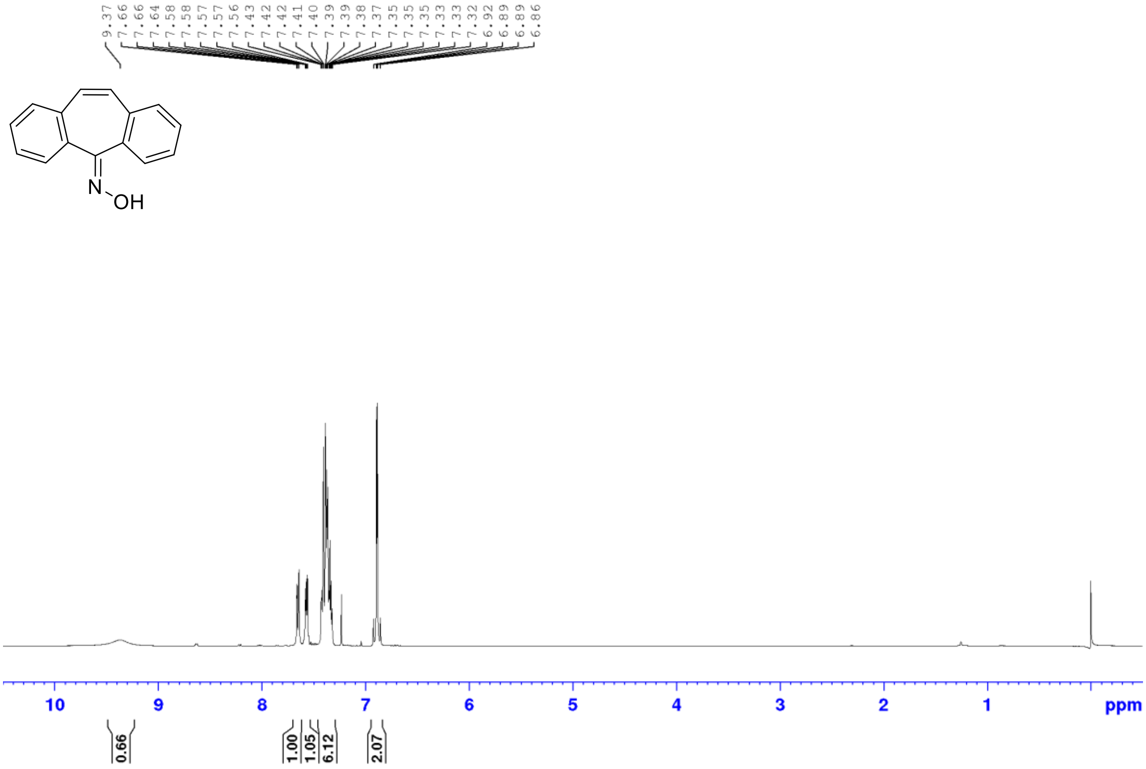

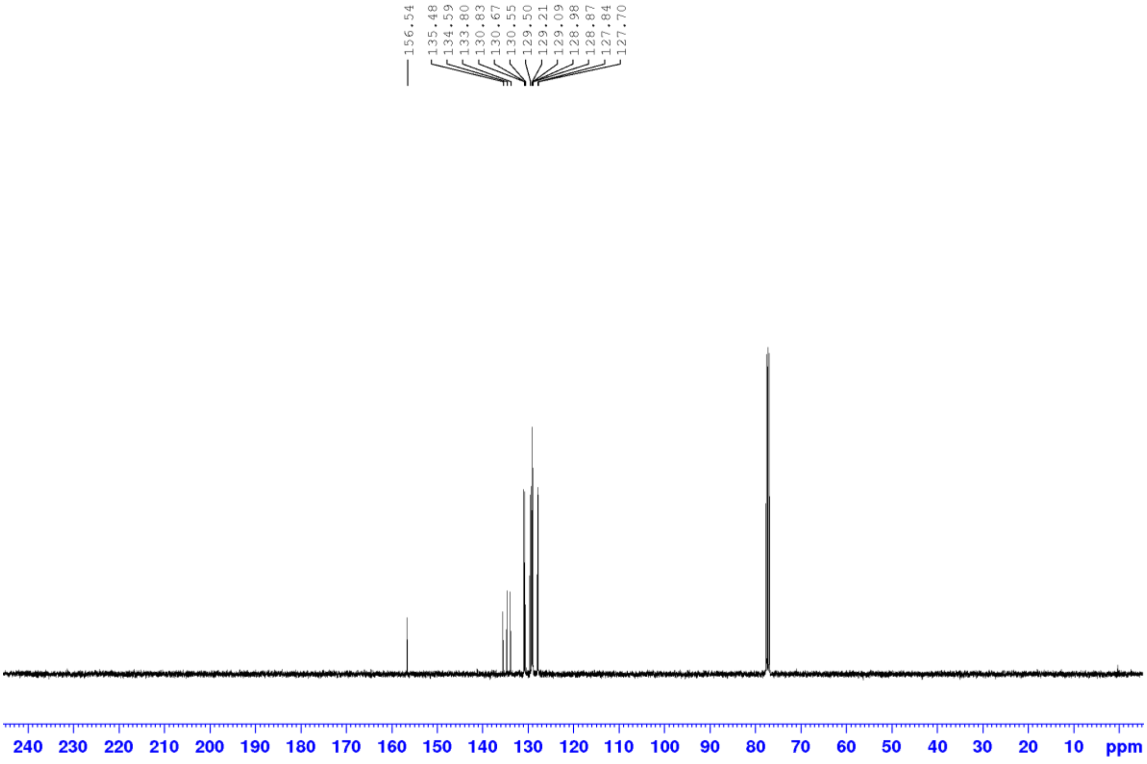

Compound 2: $CDCl_3$, 400 MHz ^1H NMR, 100 MHz ^{13}C NMR

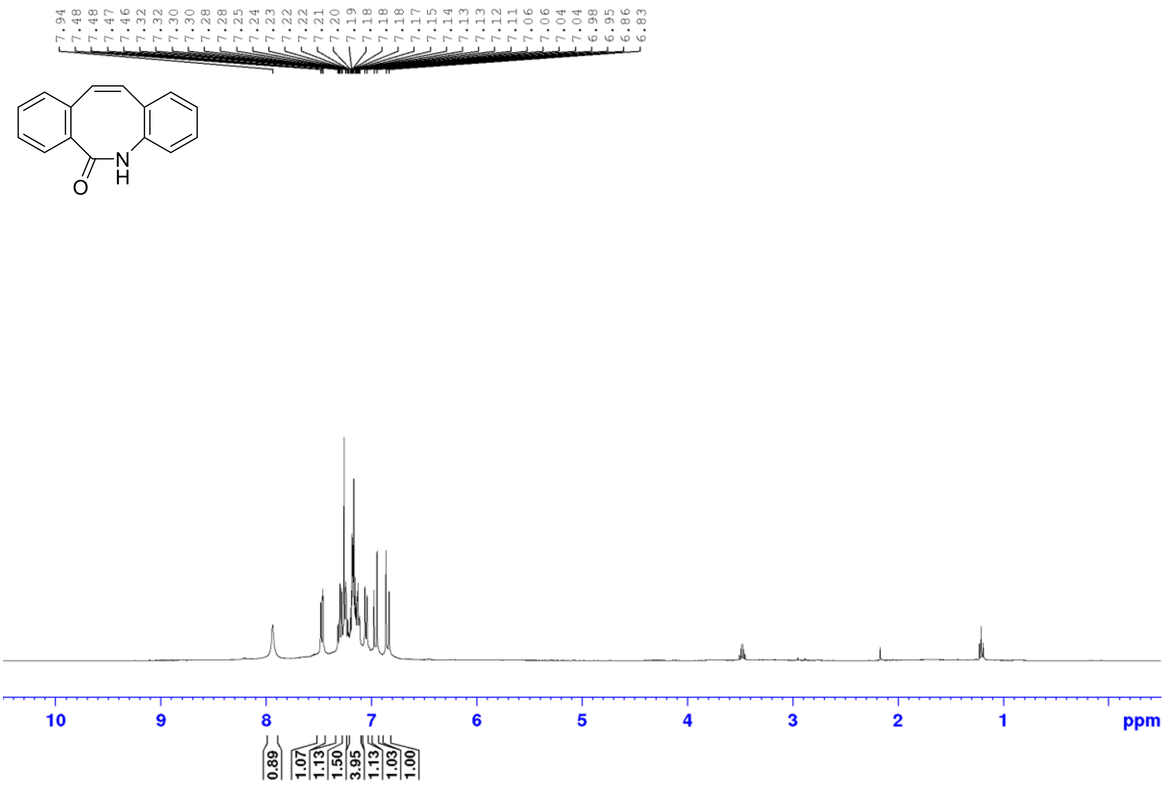

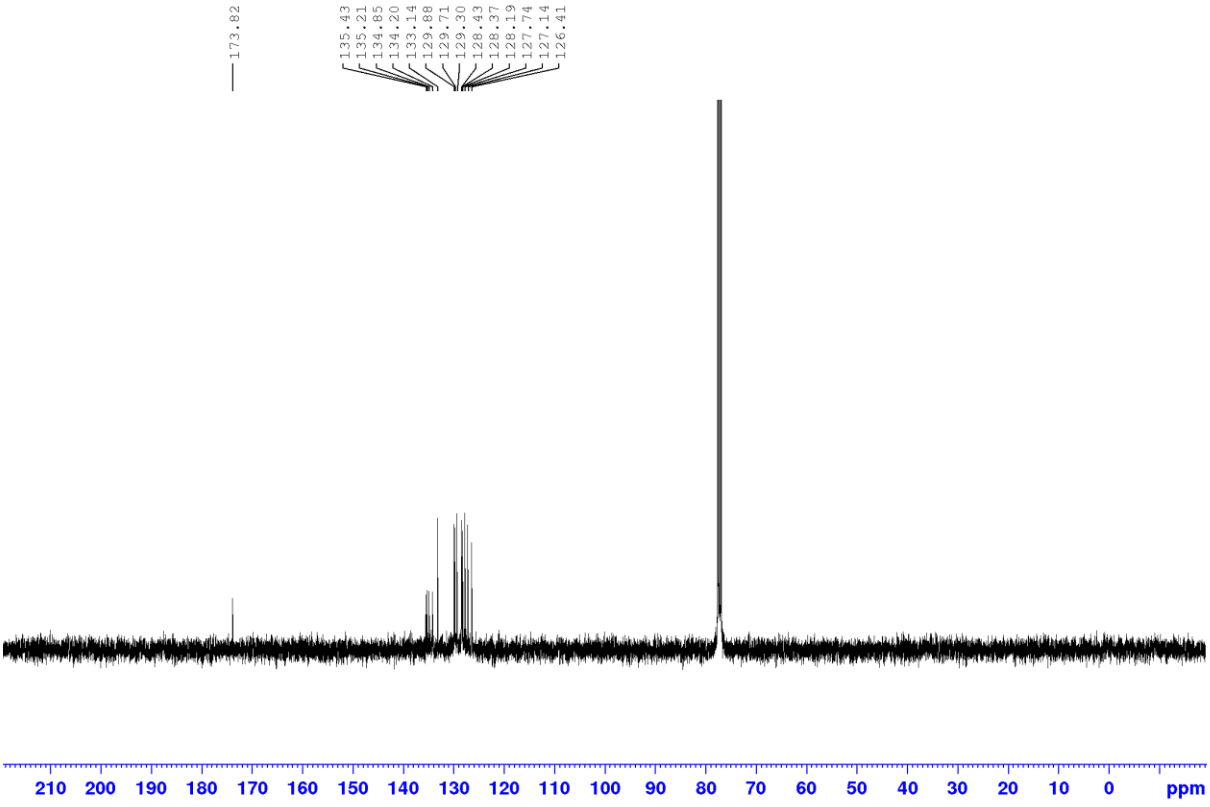

Compound 3: $CDCl_3$, 400 MHz ^{1}H NMR, 100 MHz ^{13}C NMR

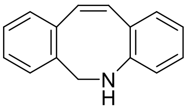

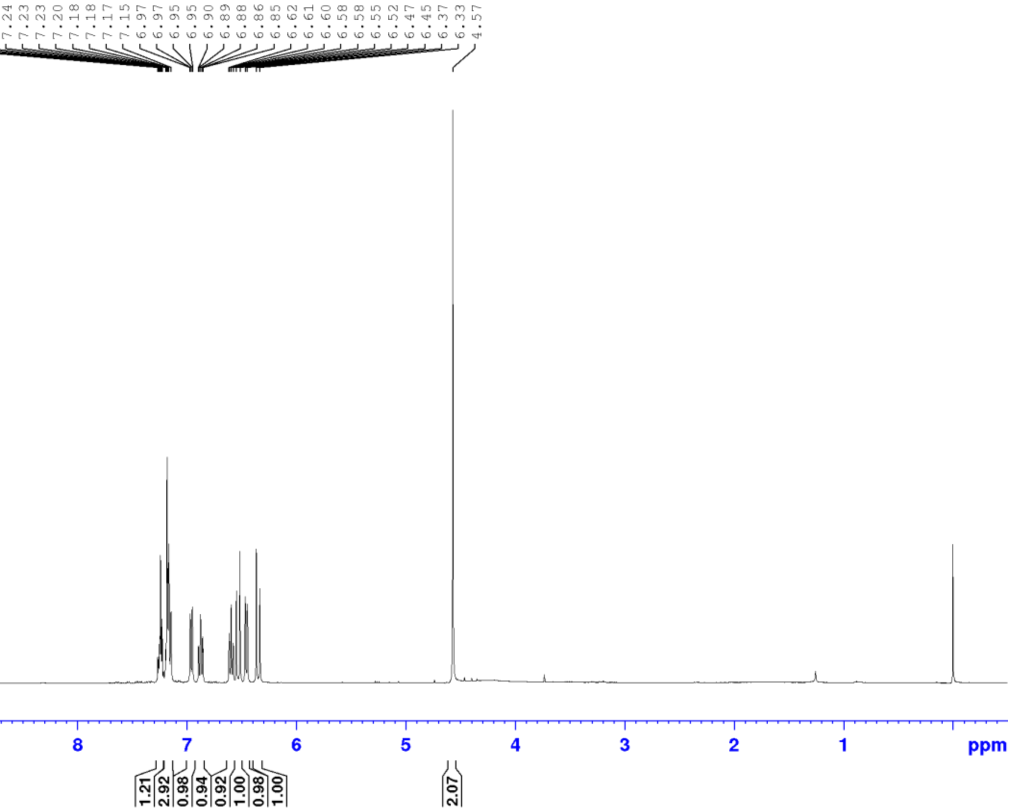

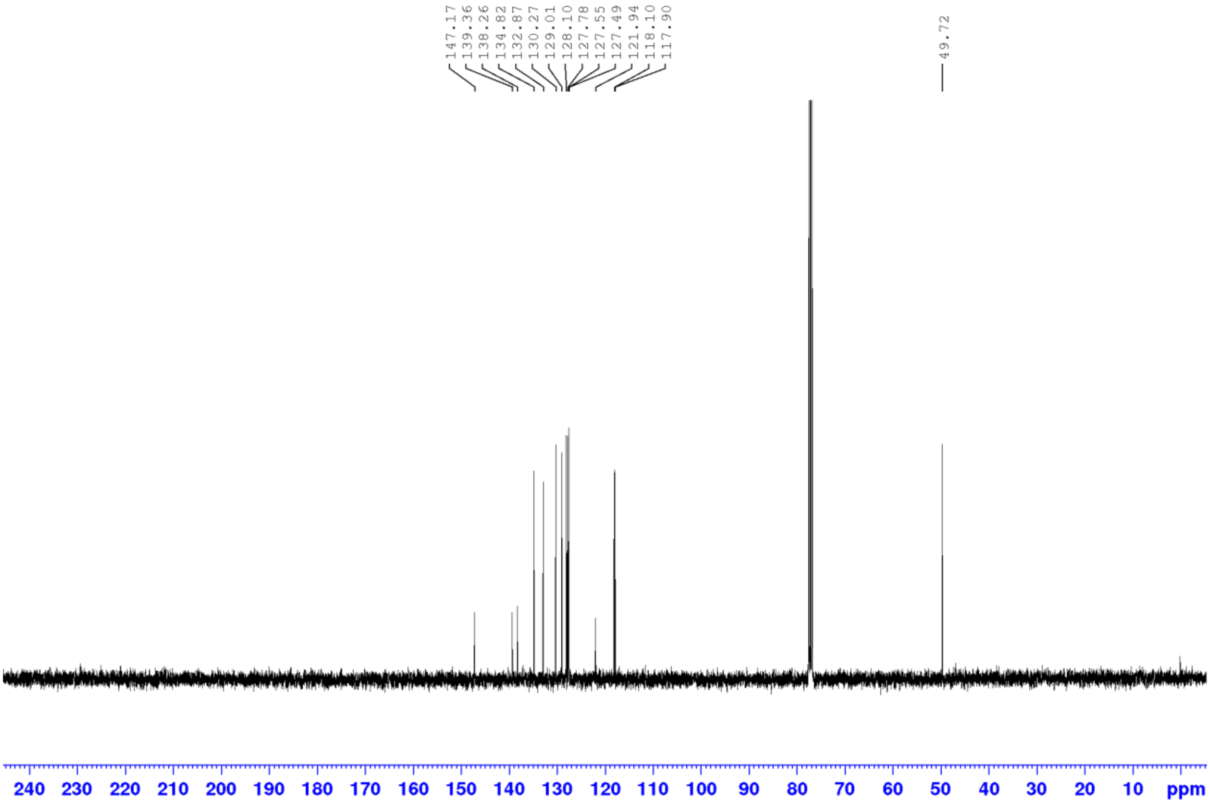

Compound 4: $CDCl_3$, 400 MHz ^1H NMR, 100 MHz ^{13}C NMR

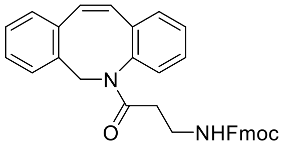

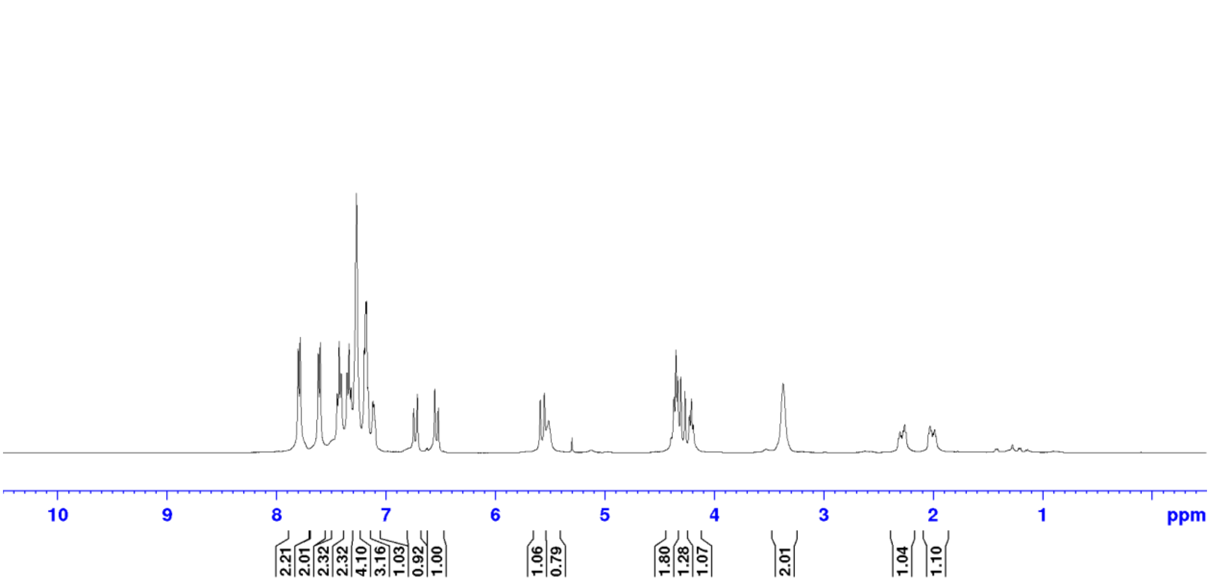

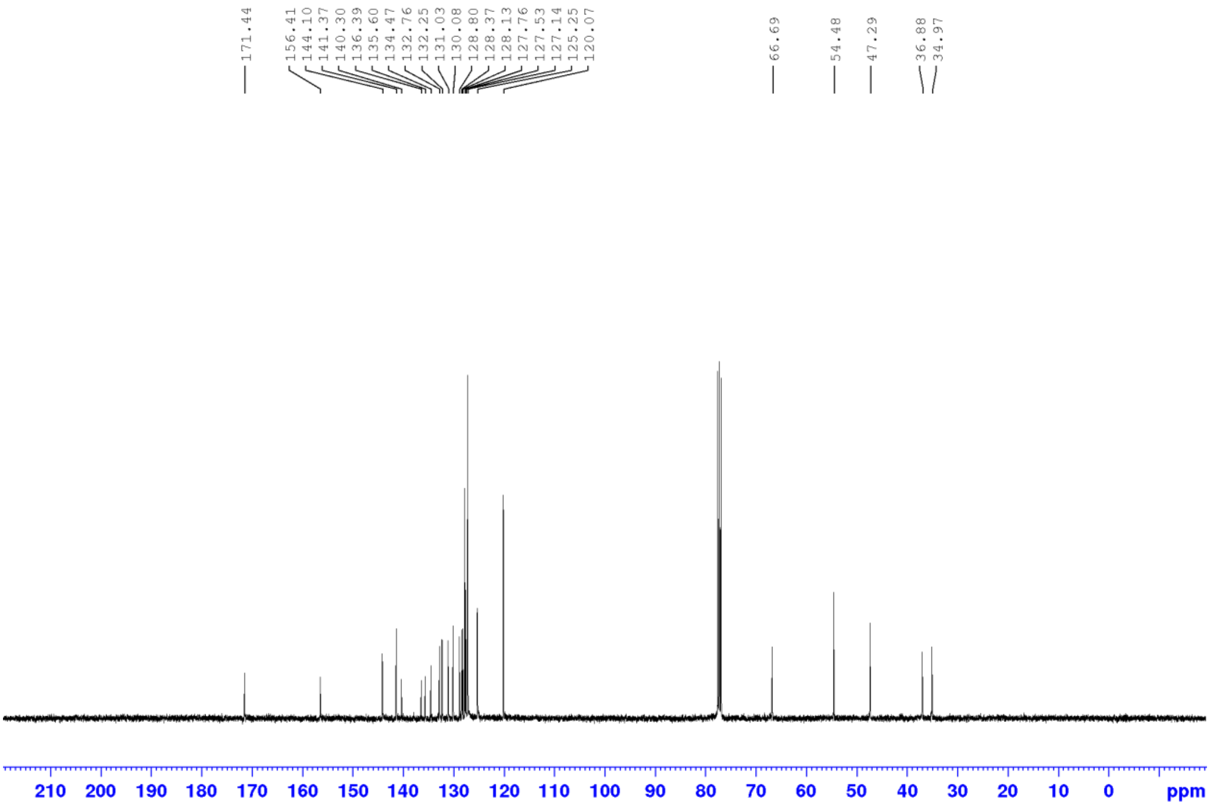

Compound 5: $CDCl_3$, 400 MHz ^{1}H NMR, 100 MHz ^{13}C NMR

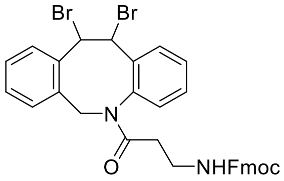

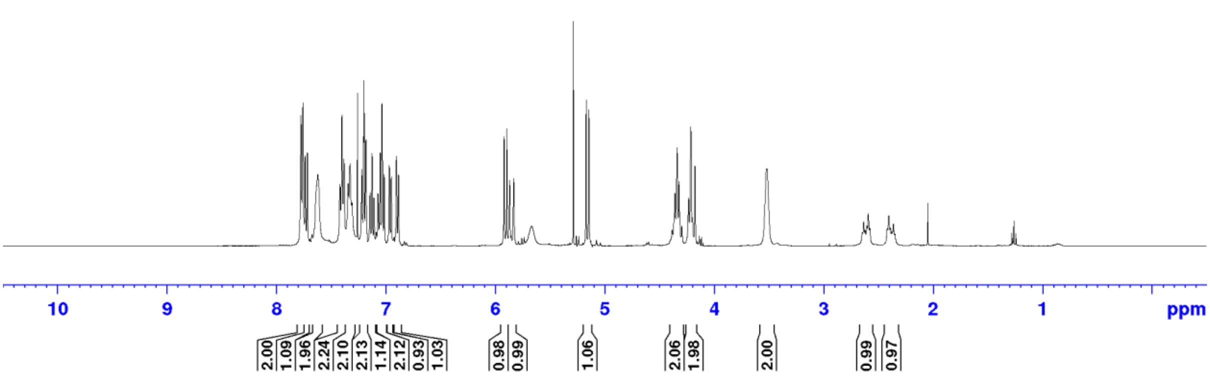

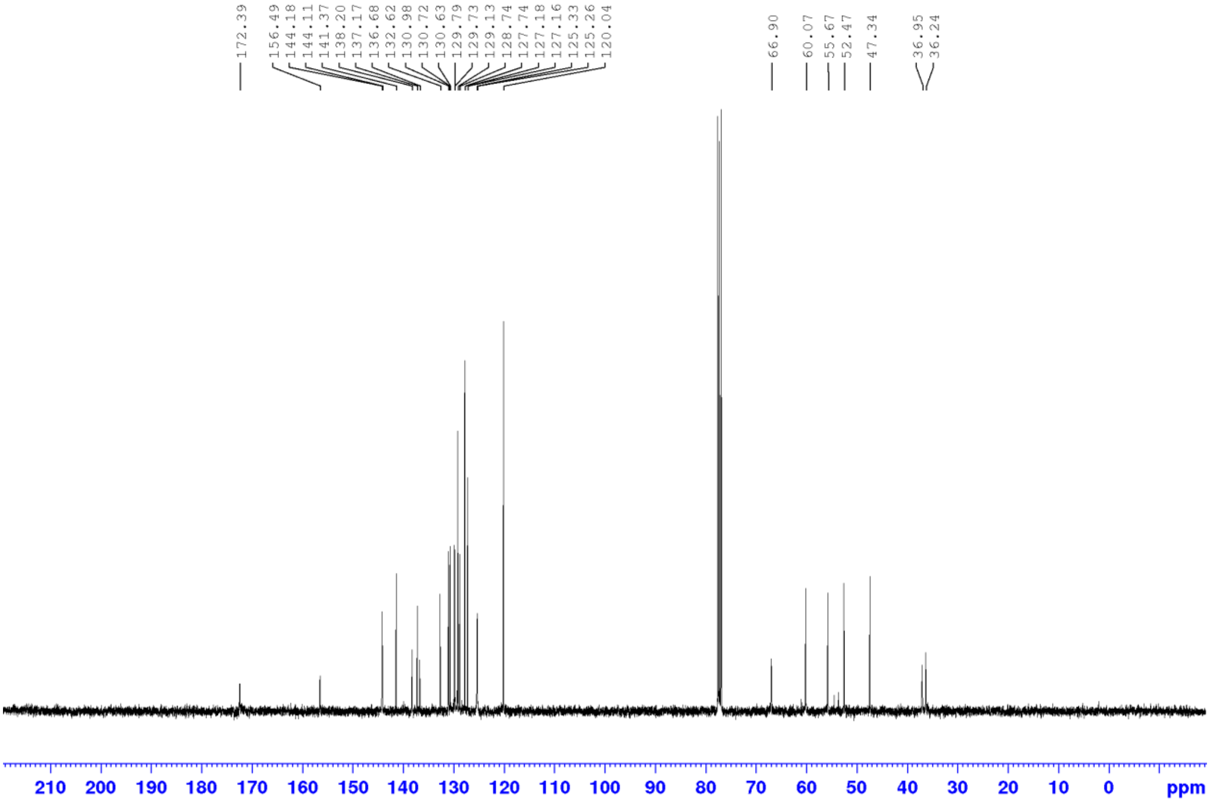

Compound 6: $CDCl_3$, 400 MHz ^1H NMR, 100 MHz ^{13}C NMR

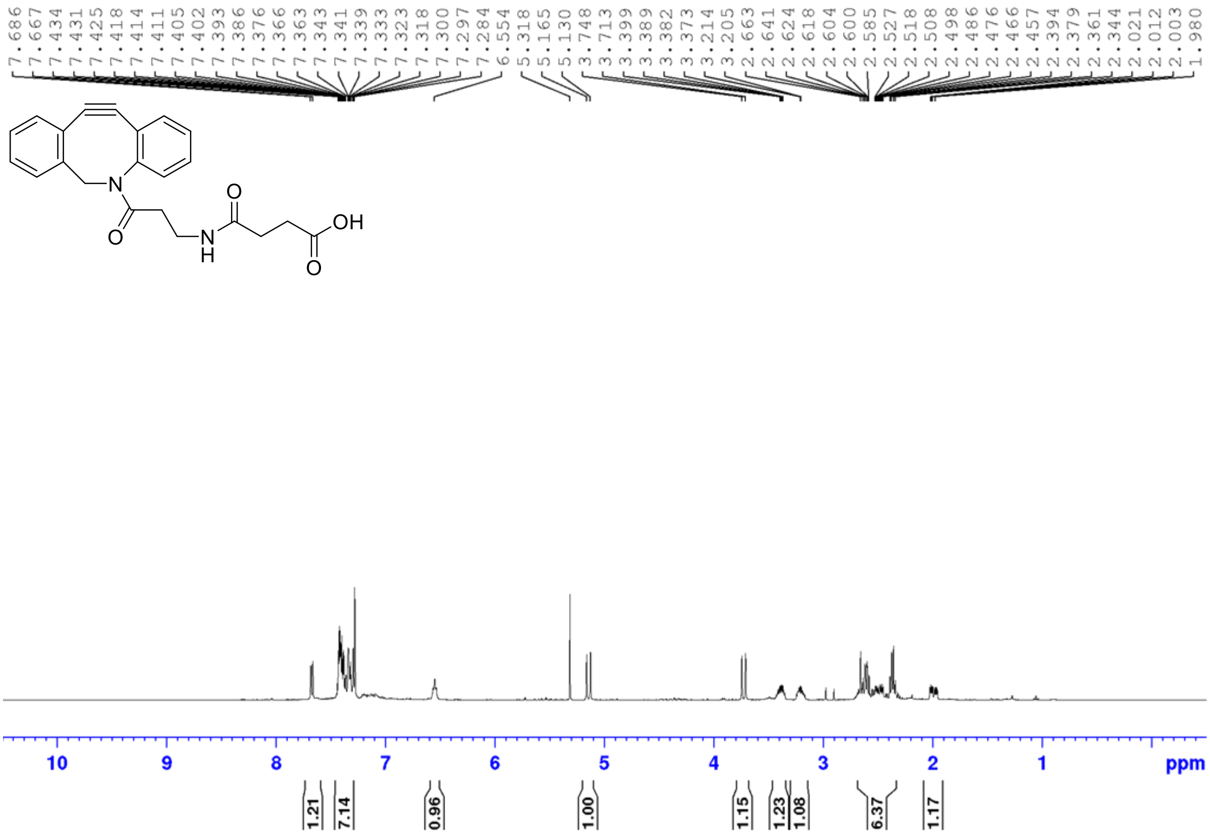

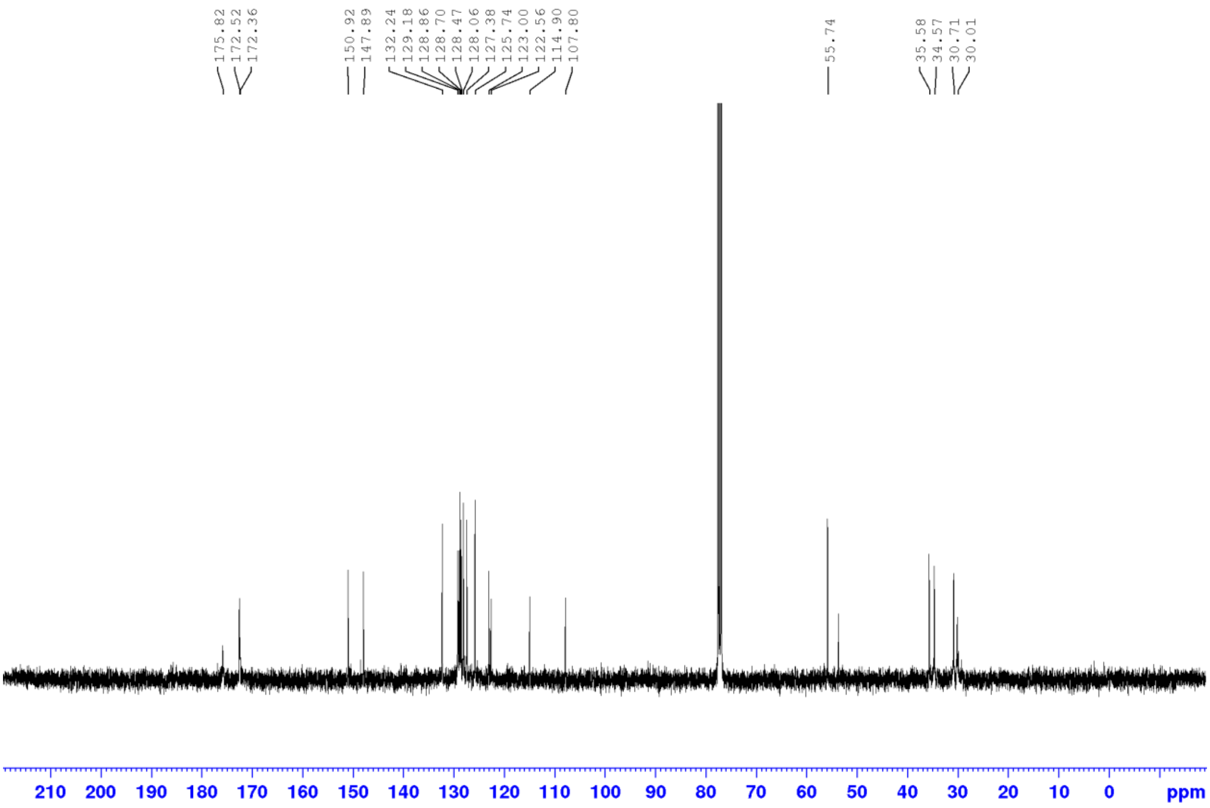

Compound 7: $CDCl_3$, 400 MHz ^1H NMR

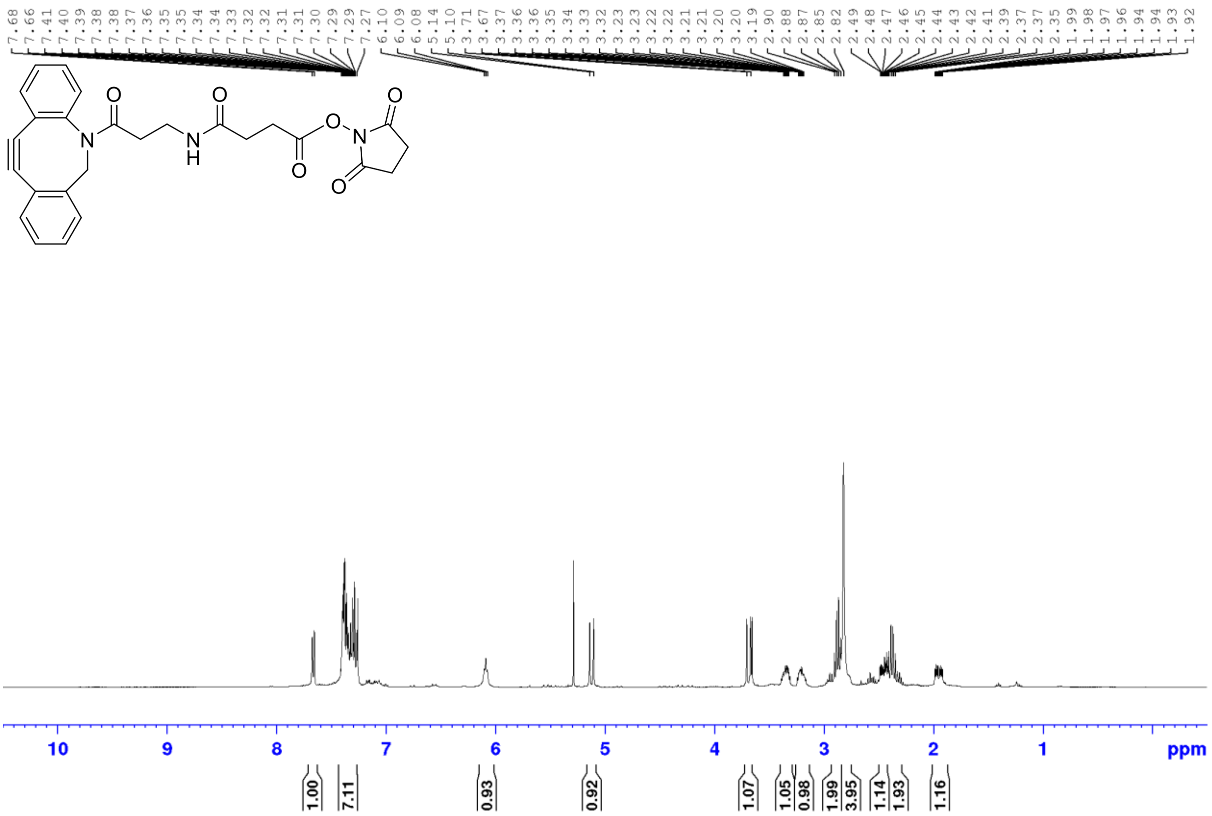

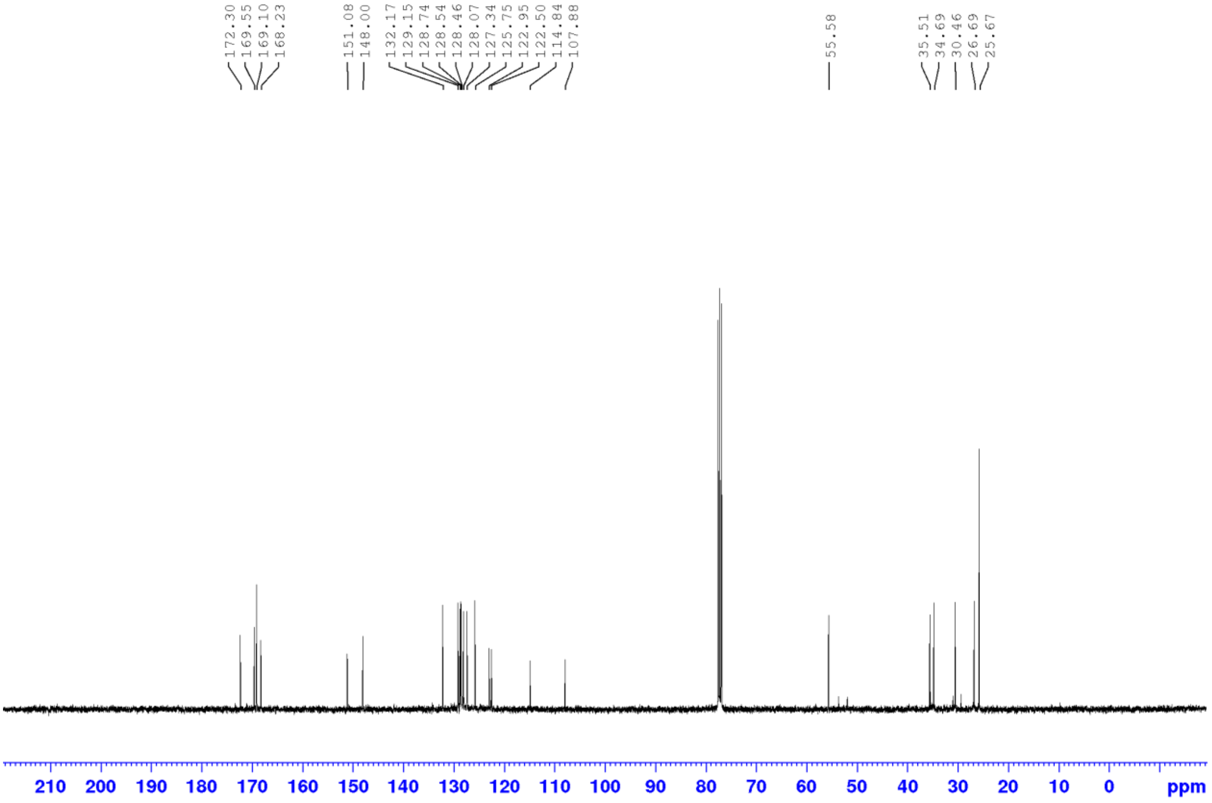

Supplement: SC-015-D3SC05054H-s001 [file SC-015-D3SC05054H-s001.pdf]
